# Comparison of inhibitory effects of irreversible and reversible Btk inhibitors on platelet function

**DOI:** 10.1002/jha2.269

**Published:** 2021-08-10

**Authors:** Bibian M.E. Tullemans, Mieke F.A. Karel, Valentine Léopold, Marieke S. ten Brink, Constance C.F.M.J. Baaten, Sanne L. Maas, Alex F. de Vos, Johannes A. Eble, Marten R. Nijziel, Emiel P.C. van der Vorst, Judith M.E.M. Cosemans, Johan W.M. Heemskerk, Theodora A.M. Claushuis, Marijke J.E. Kuijpers

**Affiliations:** ^1^ Department of Biochemistry Cardiovascular Research Institute Maastricht Maastricht University Maastricht The Netherlands; ^2^ Center for Experimental and Molecular Medicine Amsterdam University Medical Centres, Academic Medical Centre University of Amsterdam Amsterdam The Netherlands; ^3^ Hopital Lariboisiere Department of Anaesthesiology and Critical Care Paris France; ^4^ Institute for Molecular Cardiovascular Research (IMCAR) University Hospital Aachen Aachen Germany; ^5^ Interdisciplinary Center for Clinical Research (IZKF) RWTH Aachen University Aachen Germany; ^6^ Institute of Physiological Chemistry and Pathobiochemistry University of Münster Münster Germany; ^7^ Department of Haematology Catharina Hospital Eindhoven Eindhoven The Netherlands; ^8^ Department of Pathology Cardiovascular Research Institute Maastricht (CARIM) Maastricht University Medical Centre Maastricht Netherlands; ^9^ Institute for Cardiovascular Prevention (IPEK) Ludwig‐Maximilians‐University Munich Munich Germany; ^10^ Thrombosis Expertise Centre, Heart and Vascular Centre Maastricht University Medical Centre Maastricht The Netherlands

**Keywords:** bleeding, Bruton tyrosine kinase, CLEC‐2, glycoprotein VI, platelets, von Willebrand Factor

## Abstract

All irreversible Bruton tyrosine kinase (Btk) inhibitors including ibrutinib and acalabrutinib induce platelet dysfunction and increased bleeding risk. New reversible Btk inhibitors were developed, like MK‐1026. The mechanism underlying increased bleeding tendency with Btk inhibitors remains unclear. We investigated the effects of ibrutinib, acalabrutinib and MK‐1026 on platelet function in healthy volunteers, patients and Btk‐deficient mice, together with off‐target effects on tyrosine kinase phosphorylation. All inhibitors suppressed GPVI‐ and CLEC‐2‐mediated platelet aggregation, activation and secretion in a dose‐dependent manner. Only ibrutinib inhibited thrombus formation on vWF‐co‐coated surfaces, while on collagen this was not affected. In blood from Btk‐deficient mice, collagen‐induced thrombus formation under flow was reduced, but preincubation with either inhibitor was without additional effects. MK‐1026 showed less off‐target effects upon GPVI‐induced TK phosphorylation as compared to ibrutinib and acalabrutinib. In ibrutinib‐treated patients, GPVI‐stimulated platelet activation, and adhesion on vWF‐co‐coated surfaces were inhibited, while CLEC‐2 stimulation induced variable responses. The dual inhibition of GPVI and CLEC‐2 signalling by Btk inhibitors might account for the increased bleeding tendency, with ibrutinib causing more high‐grade bleedings due to additional inhibition of platelet‐vWF interaction. As MK‐1026 showed less off‐target effects and only affected activation of isolated platelets, it might be promising for future treatment.

## 1 INTRODUCTION

Ibrutinib, an irreversible Bruton's tyrosine kinase (Btk) inhibitor, is widely used to treat multiple haematological malignancies [1, 2, 3, 4, 5]. Ibrutinib treatment shows high response rates and improved progression‐free survival, with decreased toxicity when compared to conventional treatment options [1, 2, 3, 4, 5, 6]. However, ibrutinib is prescribed life‐long (or until progression) and requires side effect management. These toxicities are responsible for discontinuation in up to 20% of patients [6, 7, 8, 9]. A prominent side effect of ibrutinib is bleeding, with over 50% of patients experiencing bleeding events within 3 years, ranging from mild mucocutaneous bleeding to life‐threatening haemorrhage [3, 6, 8, 10]. Studies have suggested that ibrutinib‐associated bleeding tendency is caused by interference with platelet GPVI signalling pathway through off‐target effects on Src kinases [11, 12].

Recently, multiple other Btk inhibitors have been developed, including the irreversible Btk inhibitor acalabrutinib and the reversible Btk inhibitor MK‐1026 (formerly known as ARQ‐531) [13, 14]. Opposed to data suggesting that acalabrutinib shows less off‐target effects, it still causes an increased bleeding tendency in patients, albeit showing mainly mild bleeding [15]. Studies investigating GPVI inhibitors as potential antithrombotic drugs have shown that inhibition on receptor level does not impair haemostasis [16]. Also, GPVI‐deficient patients exhibit only a mild bleeding diathesis [17], and many potential patients are undiagnosed as they remain asymptomatic [18]. In agreement, studies in mice have shown that GPVI deficiency alone does not impair haemostasis [19, 20, 21], while combined GPVI and CLEC‐2 deficiency does [21]. Furthermore, Btk was shown to be involved in GPIb‐vWF‐mediated platelet adhesion, as well as integrin α_IIb_β_3_ outside‐in activation which were inhibited by ibrutinib [6, 22, 23]. Therefore, the current mechanism of increased bleeding tendency in patients using Btk inhibitors is still unclear and underlines the importance for treatment decisions and the development of novel Btk inhibitors. We therefore investigated the effect of multiple Btk inhibitors on platelet function pathways in healthy volunteers, patients and Btk knock‐out (KO) mice.

## 2 MATERIALS AND METHODS

Materials and methods are available in Data S1.

## 3 RESULTS

### 3.1 Ibrutinib and acalabrutinib inhibit platelet activation and aggregation by GPVI and CLEC‐2 in a dose‐dependent manner

Treatment with ibrutinib, but also acalabrutinib, can increase bleeding tendency [6, 15]. To assess the underlying pathways, we investigated platelet activation mediated by multiple agonists in presence of ibrutinib or acalabrutinib.

**FIGURE 1:**
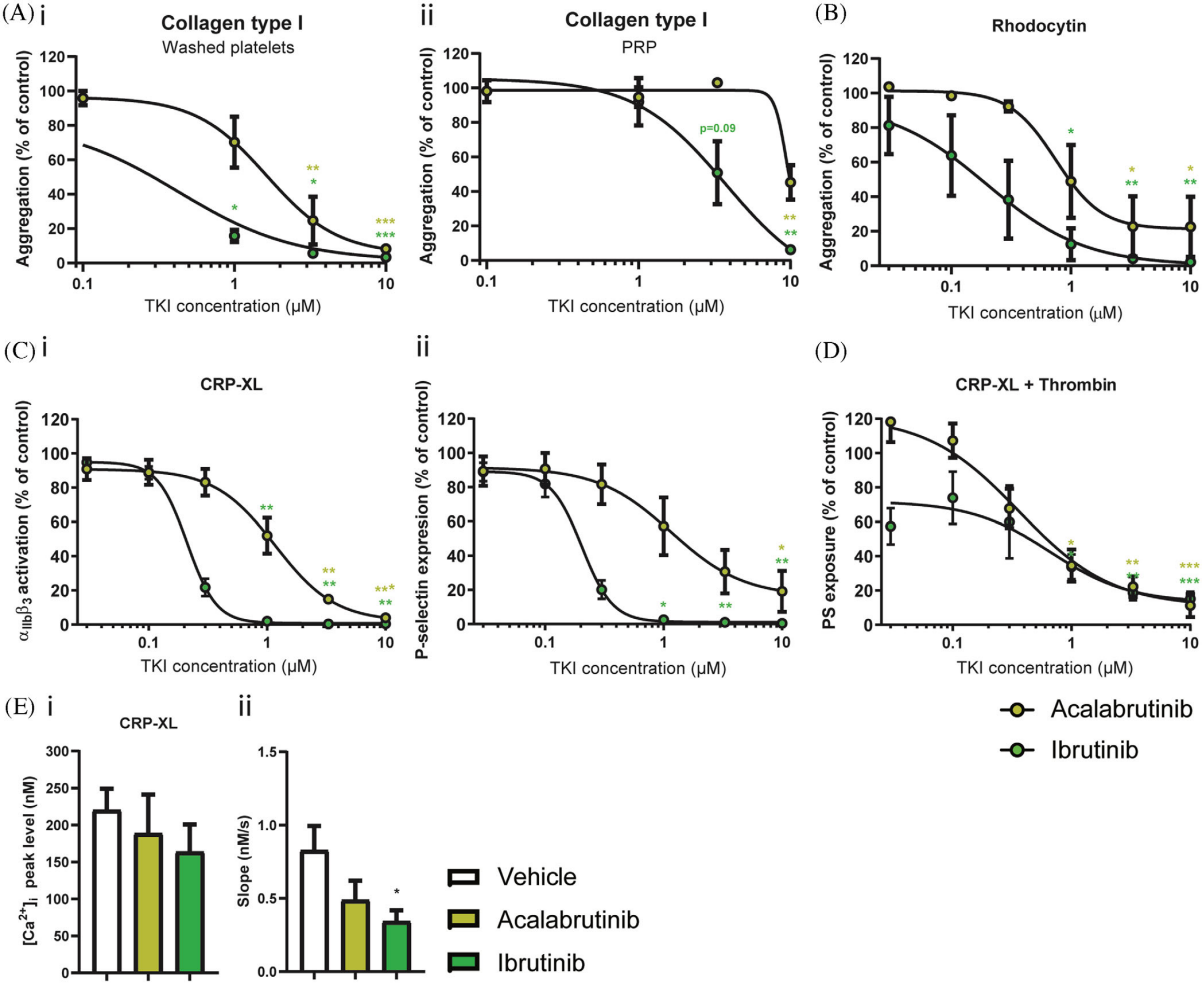
Ibrutinib and acalabrutinib inhibit GPVI‐ and CLEC‐2‐induced platelet activation and aggregation in a dose‐dependent manner. Washed platelets **(Ai**, **B**) or PRP (**Aii**) (250 × 10^9^/L) from healthy volunteers were incubated with different concentrations of acalabrutinib or ibrutinib for 10 min at 37°C. Shown are dose‐response curves of aggregation induced by collagen type I (**A**, 1 μg/ml) or rhodocytin (**B**, 1 μg/ml) in presence of acalabrutinib (lime circles) or ibrutinib (green circles) normalised to control. **(C)** Washed platelets (100 × 10^9^/L) were pre‐incubated with different concentrations of acalabrutinib or ibrutinib and stimulated with collagen‐related peptide (CRP‐XL, 5 μg/ml) for 15 min and simultaneously stained for activated integrin α_IIb_β_3_ (i) and α‐granule secretion (P‐selectin, ii). Dose response curves show the percentages of platelets binding FITC‐labelled PAC‐1 monoclonal antibody or AF647‐labelled anti‐human CD62 mAb normalized to control expression. **(D)** Platelets pre‐treated with different concentrations of acalabrutinib or ibrutinib were stimulated using both CRP‐XL (5 μg/ml) and thrombin (4 nM) for 60 min at 37°C, and PS‐exposure was determined by flow cytometry. Histogram shows percentage of platelets binding FITC‐labelled annexin A5 normalized to control expression. **(E)** Fura‐2‐loaded platelets (200 × 10^9^/L) pre‐incubated with vehicle, acalabrutinib (1 μM) or ibrutinib (1 μM) were stimulated with CRP‐XL (10 μg/ml) in presence of 2 mM CaCl_2_. Histograms show (i) maximal rise in [Ca^2+^]_i_ and (ii) slope. Data are shown as means ± SEM (*n* = 6‐7). *p < 0.05, **p < 0.01 versus control (vehicle)

Both ibrutinib and acalabrutinib inhibited aggregation of washed platelets and platelet‐rich plasma induced by 1 μg/ml collagen in a dose‐dependent manner (Figure 1A). In isolated platelets, a significant inhibition was observed at 1 μM and higher for both compounds (Figure 1Ai). In the presence of plasma, a higher concentration of 5–10 μM was required to significantly inhibit collagen‐induced platelet aggregation (Figure 1Aii). The IC_50_ values for ibrutinib and acalabrutinib were 7.7 and 6.0 times lower in washed platelets as compared to PRP (Table S1). At a higher collagen concentration, aggregation was not affected by either inhibitor. (Figure S1Aii). CLEC‐2‐induced platelet aggregation was also inhibited by both acalabrutinib and ibrutinib in a similar dose‐dependent manner (Figure 1B). GPVI‐induced integrin α_IIb_β_3_ activation, P‐selectin expression and PS‐exposure were also dose‐dependently reduced by both inhibitors, although lower concentrations of ibrutinib (<1 μM) resulted in significant inhibition as compared to acalabrutinib (Figures 1C and 1D). The IC_50_ values of ibrutinib for the inhibition of integrin α_IIb_β_3_ activation, P‐selectin expression and PS exposure were 5.0, 8.2 and 2.2 times lower, respectively, than for acalabrutinib (Table S1). Furthermore, both compounds did not alter the peak level of intracellular Ca^2+^ elevations, but the slope of this response was reduced in ibrutinib‐treated platelets (Figure 1E).

Aggregation induced by the P2Y_12_ agonist 2MeS‐ADP, the thromboxane A_2_ analogue U46619 and thrombin (Figure S1A) as well as ristocetin remained unaffected in the presence of Btk inhibitors (Figure S1B). ADP‐stimulated α_IIb_β_3_ activation and *α*‐granule secretion were mildly inhibited by both Btk inhibitors (Figure S1C). No effects on α_IIb_β_3_ activation or α‐granule secretion were observed upon thrombin activation (Figure S1C). Altogether, these results suggest that ibrutinib and acalabrutinib inhibit both GPVI‐ and CLEC‐2‐mediated platelet aggregation, albeit lower concentrations of ibrutinib were required. This might explain the increased bleeding tendency seen with both inhibitors.

### 3.2 Ibrutinib, but not acalabrutinib, impairs thrombus formation in whole blood on surfaces co‐coated with vWF

We for the first time investigated the effects of ibrutinib and acalabrutinib on whole blood thrombus formation under flow, simultaneously over multiple surfaces [24]. This allowed systematic analysis of platelet activation and thrombus formation on collagen type I and III and vWF co‐coated with laminin, rhodocytin, ristocetin or fibrinogen. In the presence of plasma, concentrations higher than 3.3 μM were required to significantly inhibit collagen‐induced aggregation (Figure 1A). Considering previous in vitro studies, as well as plasma concentrations of ibrutinib and acalabrutinib in patients for inhibition of B‐cell carcinomas [15, 25], we selected 1 μM to inhibit washed platelets and 5 μM to inhibit platelets in the presence of plasma.

**FIGURE 2:**
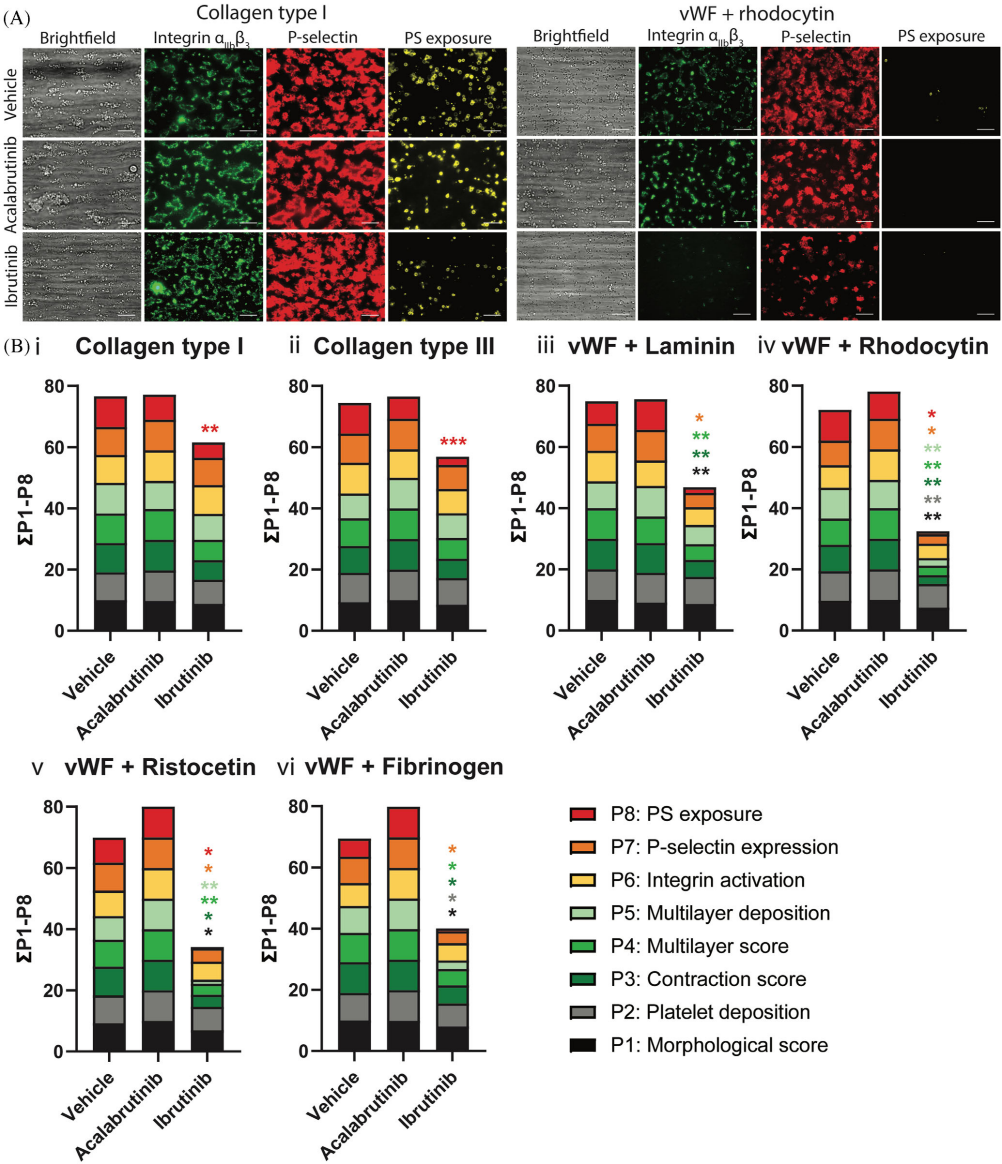
Ibrutinib, but not acalabrutinib, affected thrombus formation in whole blood perfused over six different surfaces. Pre‐incubated blood samples from healthy donors with vehicle (<0.1% DMSO), acalabrutinib (5 μM) or ibrutinib (5 μM) were recalcified in the presence of PPACK. Blood was perfused for 3.5 min at a wall shear rate of 1000 s^–1^ over six different surfaces. After perfusion, thrombi were stained with AF568‐annexin A5, FITC‐α‐fibrinogen and AF647‐α‐CD62P to detect PS‐exposure, integrin α_IIb_β_3_ activation and P‐selectin expression. **(A)** Representative images of thrombi formed on collagen type I and vWF co‐coated with rhodocytin. **(B)** Histograms show the cumulative representation of scaled values from 0 to 10 for each parameter over different surfaces consisting of (i) collagen type I, (ii) collagen type III, (iii) vWF co‐coated with laminin, (iv) vWF co‐coated with rhodocytin, (v) vWF co‐coated with ristocetin and (vi) vWF co‐coated with fibrinogen in presence of acalabrutinib or ibrutinib. Colours reflect the adhesion parameters (P1‐2; black, grey), aggregation parameters (P3‐5; shades of green) and activation parameters (P6‐8; yellow, orange and red). *p < 0.05, **p < 0.01, ***p < 0.001 versus vehicle

Microscopic visualization of platelet adhesion, activation and thrombus formation on collagen showed that neither of the two inhibitors affected these processes, except for reduced PS‐exposure by ibrutinib (Figure 2A). This was confirmed by image analysis resulting in five thrombus parameters (P1‐P5) and three activation markers (P6‐8, Figure S2). To systematically summarize the effects on all parameters, cumulative histograms were generated showing that ibrutinib, but not acalabrutinib, reduced PS‐exposure on collagen type I and III (Figure 2B, P8, decrease indicated in red). Upon blood perfusion over surfaces that trigger GPIb alone (vWF+ristocetin) or in combination with CLEC‐2 (vWF+rhodocytin), α_6_β_1_ (vWF+laminin) or α_IIb_β_3_ (vWF+fibrinogen), only ibrutinib decreased almost all parameters of thrombus formation (Figures 2B and S2). In comparison to control, thrombi were smaller and less compact in structure with ibrutinib and showed reduced expression of activated integrin α_IIb_β_3_ and P‐selectin (*α*‐granule release) (Figure S2). These results show that, in contrast to acalabrutinib, platelet adhesion to surfaces co‐coated with vWF was strongly impaired by ibrutinib.

### 3.3 Effects of ibrutinib and acalabrutinib on wild‐type and Btk‐deficient mouse platelets

**FIGURE 3:**
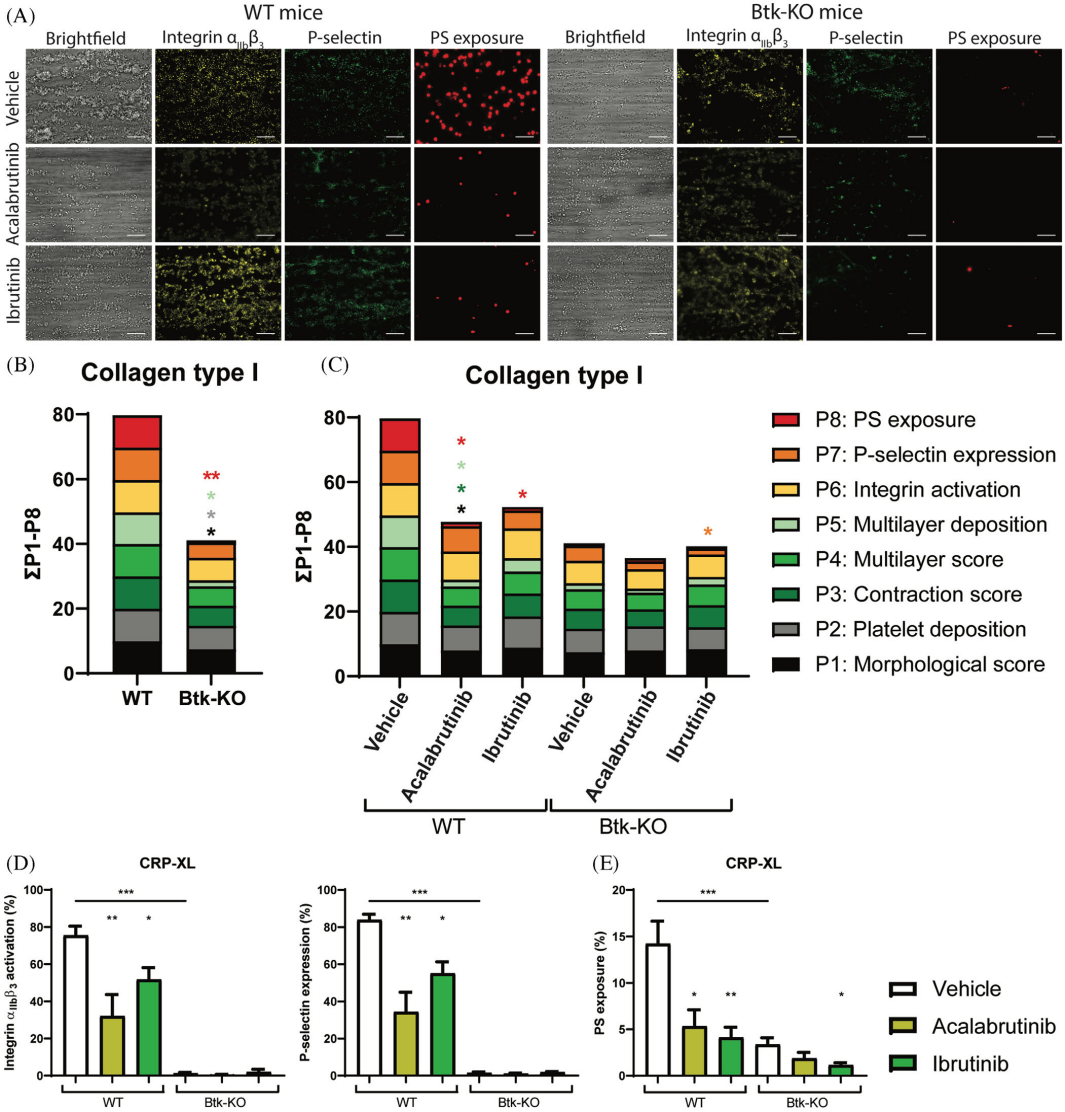
Acalabrutinib and ibrutinib reduced thrombus formation and PS‐exposure in whole blood under flow, as well as activation of isolated platelets from WT but not Btk‐KO mice. Pooled citrated whole blood samples from two wildtype (WT) or Btk‐KO mice were pre‐incubated with vehicle (<0.1% DMSO), acalabrutinib (5 μM) or ibrutinib (5 μM) in the presence of PPACK and fragmin for 10 min at 37°C. After incubation, blood samples were recalcified and perfused for 3.5 min at a wall shear rate of 1000 s^–1^ over a collagen type I surface. Thrombi were post‐stained with FITC‐labelled anti‐CD62P mAb, PE‐labelled JonA mAb and AF647‐labelled Annexin‐A5 to detect P‐selectin expression, integrin activation and PS‐exposure, respectively. **(A)** Representative brightfield and fluorescence images from thrombus formation in blood from WT and Btk‐KO mice for each inhibitor. **(B)** Histograms show the cumulative representation of scaled values from 0 to 10 for each parameter on a collagen type I surface of thrombus formation in WT and Btk‐KO mice blood samples. Colours reflect the adhesion parameters (P1‐2; black, grey), aggregation parameters (P3‐5; shades of green) and activation parameters (P6‐8; yellow, orange and red). **(C)** Histograms of cumulative scaled values of multiple parameters in WT or Btk‐KO mice with addition of vehicle (<0.1% DMSO), acalabrutinib or ibrutinib. **(D)** Platelet activation in whole blood induced by CRP‐XL (1 μg/ml). Histograms show the percentages of platelets binding PE‐labelled JON/A monoclonal antibody against activated α_IIb_β_3_ integrin or BB700‐labelled CD62P mAb in WT and Btk‐KO mice. **(E)** PS‐exposure of platelets induced by CRP‐XL (1 μg/ml). Histogram shows percentage of platelets binding FITC‐labelled lactadherin. Data are shown as means ± SEM (*n* = 6‐8). *p < 0.05. **p < 0.01, ***p < 0.001 versus vehicle or otherwise indicated

To further examine the effects of ibrutinib and acalabrutinib on platelets, thrombus formation and platelet activation were determined in blood from wild‐type (WT) and Btk‐KO mice. Blood samples were incubated with ibrutinib or acalabrutinib before perfusion over collagen type I. In control conditions (vehicle), thrombus formation was reduced in blood from Btk‐KO mice with regard to platelet deposition, multilayer formation (P1‐2, P5) and PS‐exposure (P8, Figures 3A, 3B and S3A). In WT blood, the presence of acalabrutinib gave a similar inhibitory pattern of thrombus formation (Figures 3A and 3C). Comparable to human blood, incubation of WT mouse blood with ibrutinib resulted in limited effects on thrombus formation, only a decrease in PS‐exposure was observed (Figures 3A, 3C and S3A). When Btk inhibitors were pre‐incubated in blood from Btk‐KO mice, no additional inhibition on thrombus formation was observed (Figures 3A, 3C and S3A). The only observed effect was a reduction in P‐selectin expression in the presence of ibrutinib, as compared to vehicle, in Btk‐KO mice (Figures 3A, 3C and S3).

GPVI‐induced platelet activation by CRP‐XL was strongly reduced in Btk‐KO platelets as compared to WT with regard to α_IIb_β_3_ activation, α‐granule release and PS‐exposure, as determined by flow cytometry (Figures 3D and 3E). This was in agreement with the results obtained from whole blood perfusion experiments. Interestingly, α_IIb_β_3_ activation and P‐selectin expression were also moderately reduced in Btk‐KO mice upon PAR4 stimulation, while activation with ADP was not affected (Figures S3B and S3C). When blood was preincubated with Btk inhibitors, we observed significant reduction of all platelet activation markers in WT platelets upon GPVI stimulation (Figures 3D and 3E). In platelets from Btk‐KO mice, both inhibitors did not further reduce α_IIb_β_3_ activation and P‐selectin expression regardless of the agonist. We only observed significant reduction of PS‐exposure in Btk‐KO platelets in the presence of ibrutinib, as compared to vehicle (Figure 3E).

Overall, preincubation of whole blood from Btk‐KO mice with acalabrutinib and ibrutinib did not result in major additional effects on thrombus formation on collagen under flow, as well as on platelet activation.

### 3.4 Btk inhibitors reduce GPVI‐induced phosphorylation of multiple platelet tyrosine kinases

**FIGURE 4:**
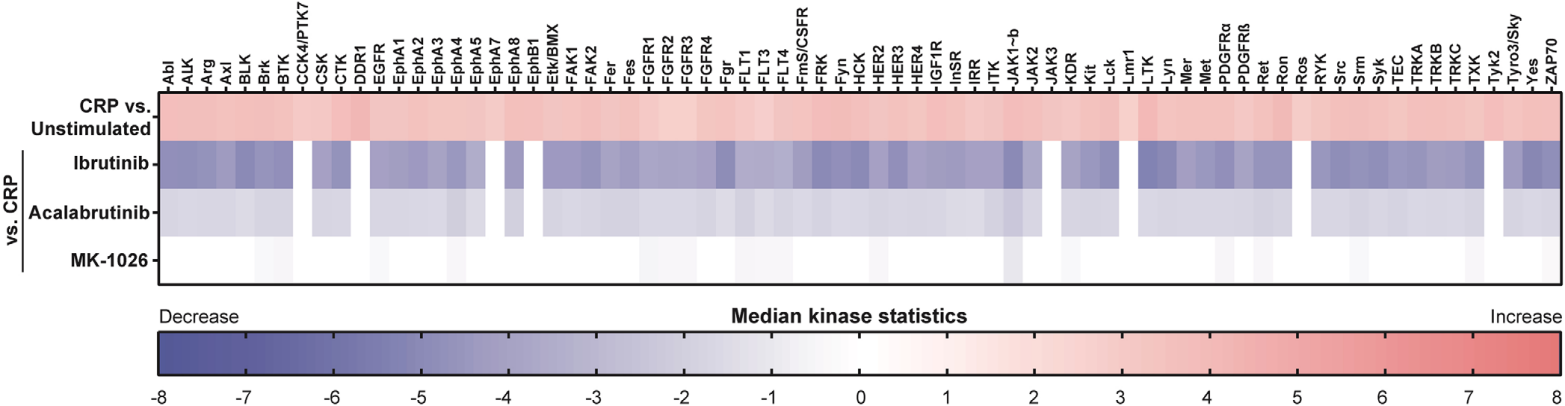
Effect of ibrutinib, acalabrutinib and MK‐1026 on GPVI‐induced tyrosine kinase activity. Washed platelets (500 × 10^9^/L) were pretreated with vehicle (control) or 1 μM ibrutinib, acalabrutinib or MK‐1026 for 10 min at 37°C and were subsequently stimulated for 90 s with 5 μg/ml CRP‐XL in the presence of 2 mM CaCl_2_. Unstimulated, resting platelets served as control. Heatmap visualizes all tyrosine kinases that are significantly up‐ or downregulated upon CRP stimulation (sorted alphabetically). Cut‐off has been set at a median final score of at least 1.2. Colour reflects the calculated median kinase statistic reflecting effect size and directionality (red: upregulated; blue: downregulated)

To evaluate off‐target effects of the Btk inhibitors, we performed a PamGene kinase assay to visualize which tyrosine kinases were regulated upon platelet stimulation via GPVI, as well as those tyrosine kinases that were subsequently influenced upon pre‐incubation of the platelets with 1 μM ibrutinib or acalabrutinib. We observed that in isolated platelets, in total 73 tyrosine kinases were significantly phosphorylated by stimulation with CRP, 65 of which were significantly inhibited by ibrutinib and acalabrutinib preincubation (Figure 4). Median kinase statistics showed that ibrutinib inhibited tyrosine phosphorylation of these proteins on average 4.4‐fold, while acalabrutinib was less strong and reduced this response 1.8‐fold, that is, 2.5‐fold weaker as compared to ibrutinib.

In comparison to these irreversible inhibitors, we investigated if the reversible Btk inhibitor MK‐1026^[14]^ showed a more favourable anti‐platelet effect. First, we evaluated which platelet tyrosine kinases were affected by this compound upon GPVI stimulation. Only 18 tyrosine kinases were significantly inhibited by MK‐1026 (Figure 4). Median kinase statistics showed that MK‐1026 inhibited tyrosine phosphorylation of these proteins on average 0.52‐fold, which was 8.3‐fold lower as compared to ibrutinib. As expected, all three inhibitors significantly inhibited the phosphorylation of Btk, and ibrutinib and acalabrutinib also inhibited Tec. Furthermore, ibrutinib and acalabrutinib also inhibited the phosphorylation of Src family kinases and other downstream proteins as previously described [11, 12].

These results show that ibrutinib and acalabrutinib showed the same off‐target effects on GPVI‐induced tyrosine kinase phosphorylation in platelets, although the effects of acalabrutinib were less strong. The reversible inhibitor MK‐1026 appeared to have less off‐target effects in platelets upon GPVI stimulation, in combination with a less strong inhibition profile as compared to the irreversible inhibitors.

### 3.5 The reversible Btk inhibitor MK‐1026 shows only limited effects on human and mouse platelets in vitro

**FIGURE 5:**
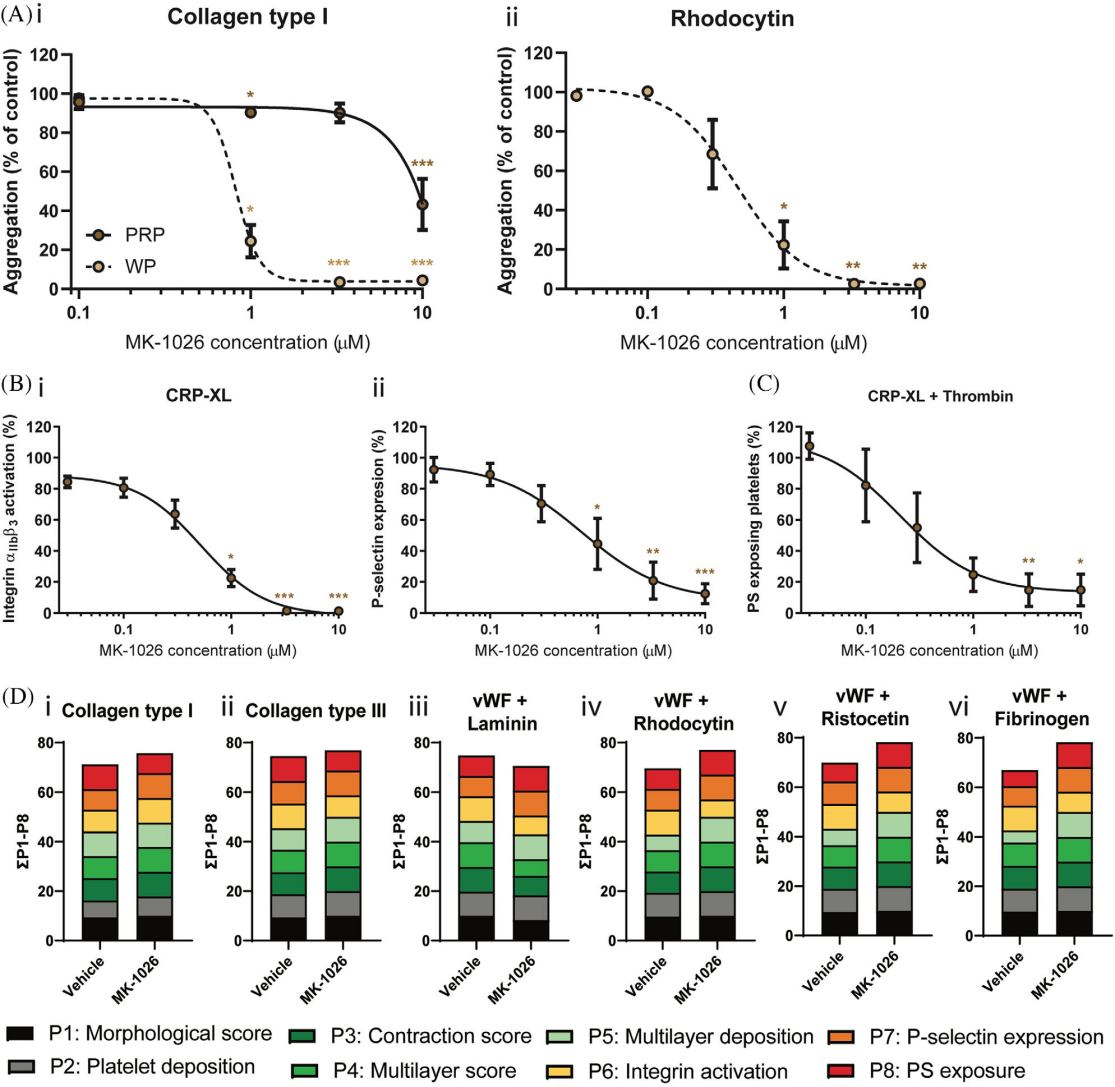
Effects of MK‐1026 on aggregation and activation of isolated platelets in response to several agonists as well as whole blood thrombus formation under flow. **(A)** PRP or washed platelets (250 × 10^9^/L) from healthy volunteers were incubated with different concentrations of MK‐1026 for 10 min at 37°C. Shown are dose‐response curves of aggregation induced by (i) collagen type I (1 μg/ml) or (ii) rhodocytin (1 μg/ml) in the presence of MK‐1026 normalised to control expression. **(B)** Washed platelets (100 × 10^9^/L) were pre‐incubated with different concentrations of MK‐1026 and stimulated with collagen‐related peptide (CRP‐XL, 5 μg/ml) for 15 min and simultaneously stained for activated integrin α_IIb_β_3_ and α‐granule secretion (P‐selectin). Histograms show the percentages of platelets binding FITC‐labelled PAC‐1 monoclonal antibody or AF647‐labelled anti‐human CD62 mAb normalized to control expression. **(C)** Platelets pre‐treated with different concentrations of MK‐1026 were stimulated using both CRP‐XL (5 μg/ml) and thrombin (4 nM) for 60 min at 37°C, and PS‐exposure was determined by flow cytometry. Histogram shows percentage of platelets binding FITC‐labelled annexin A5 normalized to control expression. Data are shown as means ± SEM (*n* = 6–7). **(D)** Pre‐incubated blood samples from healthy donors with vehicle (<0.1% DMSO) or MK‐1026 (5 μM) were recalcified in the presence of PPACK. Blood was perfused for 3.5 min at wall shear rate of 1000 s^–1^ over six different coated surfaces. Thrombi were post‐stained with AF568‐annexin A5, FITC‐α‐fibrinogen and AF647‐α‐CD62P to detect PS‐exposure, integrin activation and P‐selectin expression. Cumulative histograms represent the scaled values (0–10) for each of the parameters. Colours reflect the adhesion parameters (P1‐2; black, grey), aggregation parameters (P3‐5; shades of green) and activation parameters (P6‐8; yellow, orange and red). *p < 0.05, **p < 0.01, ***p < 0.001 versus vehicle

As we demonstrated that irreversible inhibition of Btk can impair platelet function, we also investigated the effects of the reversible Btk inhibitor MK‐1026 on platelet function. First, we performed collagen‐ and rhodocytin‐induced dose‐response aggregation experiments in PRP and washed platelets. Similar to the irreversible Btk inhibitors, MK‐1026 inhibited platelet aggregation (with more prominent effects in washed platelets compared to PRP) at multiple concentrations induced by both agonists (Figure 5A). GPVI‐induced integrin α_IIb_β_3_ activation, P‐selectin expression and PS‐exposure were also dose‐dependently reduced by MK‐1026 (Figure 5B). The calculated IC_50_ values for MK‐1026 in the presence of plasma were similar as for acalabrutinib (Table S1). Based on these results, in combination with reported in vivo plasma concentrations [26], we selected 5 μM for further experiments. Aggregation induced by ADP and U46619 were slightly but significantly reduced at this concentration of MK‐1026, while responses to thrombin and ristocetin were unaffected (Figures S4A and S4B). Integrin α_IIb_β_3_ activation and P‐selectin expression were not inhibited by MK‐1026 in platelets stimulated with ADP or thrombin (Figure S4C). Also, MK‐1026 did not alter platelet adhesion and thrombus formation on six different surfaces, as seen with acalabrutinib (Figures 5D and S5).

**FIGURE 6:**
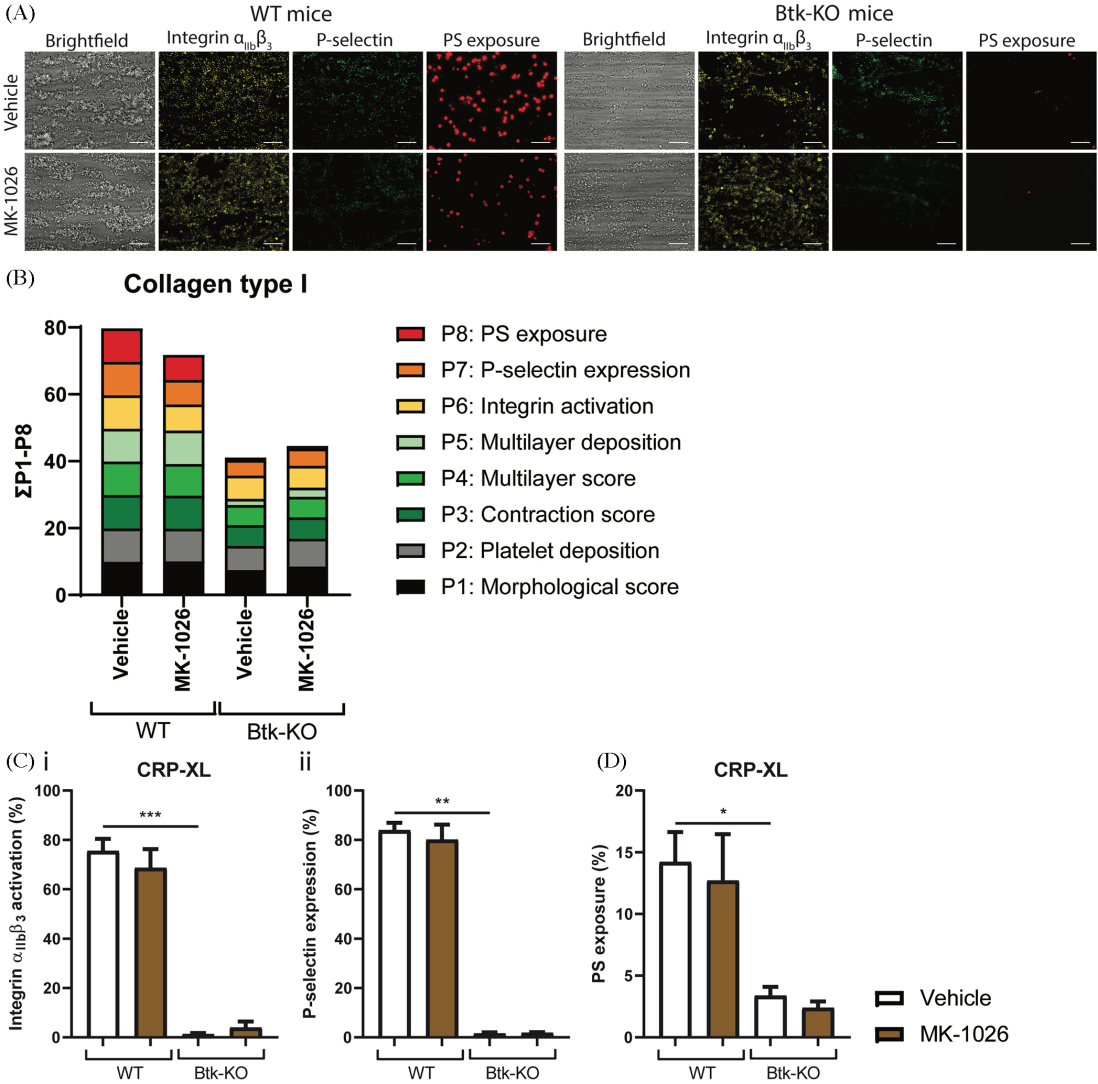
Thrombus formation, platelet activation and PS‐exposure in blood from WT and Btk‐KO mice were unaffected by MK‐1026. Pooled citrated whole blood samples from two wild‐type (WT) or Btk‐KO mice were pre‐incubated with vehicle (<0.1% DMSO) or MK‐1026 (5 μM) in the presence of PPACK and fragmin for 10 min at 37°C. After incubation, blood samples were recalcified and perfused for 3.5 min at a wall shear rate of 1000 s^–1^ over a collagen type I surface. Thrombi were post‐stained with FITC‐labelled anti‐CD62P mAb, PE‐labelled JON/A mAb and AF647‐labelled Annexin‐A5. **(A)** Representative brightfield and fluorescence images from thrombus formation in blood from WT and Btk‐KO mice. **(B)** Histograms of cumulative scaled values of multiple parameters in WT or Btk‐KO mice with addition of vehicle (<0.1% DMSO) or MK‐1026. Colours reflect the adhesion parameters (P1‐2; black, grey), aggregation parameters (P3‐5; shades of green) and activation parameters (P6‐8; yellow, orange and red). **(C)** Platelet activation in whole blood induced by CRP‐XL (1 μg/ml). Histograms show the percentages of platelets binding PE‐labelled JON/A monoclonal antibody or BB700‐labelled CD62P mAb in WT and Btk‐KO mice. **(D)** PS‐exposure of platelets induced by CRP‐XL (1 μg/ml). Histogram shows percentage of platelets binding FITC‐labelled lactadherin. Data are shown as means ± SEM (*n* = 6‐8). *p < 0.05, **p < 0.01, ***p < 0.001

We also investigated the effects of MK‐1026 using blood from Btk‐KO mice. Similarly as for acalabrutinib and ibrutinib, we did not observe additional inhibition of whole blood thrombus formation on collagen with MK‐1026 in blood samples from WT or Btk‐KO mice (Figures 6A, 6B and S6A). Also, no inhibitory effect of MK‐1026 was found upon platelet activation or PS‐exposure from WT or Btk‐KO mice with CRP‐XL, ADP or PAR4 agonist (Figures 6C, 6D, S6B and S6C).

These data show that the reversible Btk inhibitor MK‐1026 inhibited GPVI‐ and CLEC‐2‐mediated aggregation of washed platelets but did not impair platelet adhesion or activation in whole blood perfused over collagen.

### 3.6 Ibrutinib inhibits thrombus formation under flow on multiple surfaces and GPVI‐induced platelet activation responses in patients

Next, we assessed which platelet activation pathways were altered in patients using ibrutinib. Sixteen patients were included, of which seven did not receive ibrutinib, and nine were treated with 420–560 mg ibrutinib once a day (Table 1). Baseline characteristics were largely similar between groups. Importantly, none of the patients were thrombocytopenic, except one patient with a platelet count of 50 × 10^9^/L (Table 1). Of the ibrutinib‐treated patients, 66% (6/9) reported bleeding symptoms with a median ISTH‐BAT score of 2 (range 1–7, Table 1). Of patients without ibrutinib treatment, none reported bleeding symptoms.

**TABLE 1:** Characteristics of the patients

						**Haematological parameters**			**Bleeding**	
**Group**	**Gender**	**Age (years)**	**Disease**	**Ibrutinibtreatment (mg/d)**	**Anti‐platelet drugs**	**WBC (x10^9^/L)**	**RBC (x10^12^/L)**	**PLT (x10^9^/L)**	**Yes/No**	**ISTH BAT score**
**Patients**	Male	73	WM	–	–	3.6	4.3	140	No	0
	Male	45	WM	–	–	6.9	3.7	200	No	0
	Male	68	CLL	–	–	7.7	4.2	190	No	0
	Female	74	CLL	–	–	48.5	3.6	160	No	0
	Male	86	MCL	–	ASA, CLOP	6.8	3.8	160	No	0
	Male	65	WM	–	–	4.4	3.6	220	No	0
	Male	64	MCL	–	ASA	3.9	4.1	200	No	0
Median (±IQR)		68 (64–74)		–	–	6.8 (3.9–7.7)	3.8 (3.6–4.2)	190 (160–200)	0	
**Patients + ibrutinib**	Female	75	DLBCL leg type	560	–	5.7	4.5	380	Yes	1
	Male	90	MCL	560	–	0.9	2.5	50	Yes	1
	Female	66	CLL	420	–	3.9	4.3	160	Yes	3
	Male	51	WM	420	–	6.9	3.7	210	No	0
	Female	62	WM	420	–	4.5	4.0	180	No	0
	Male	80	MCL	280	–	8.6	3.5	240	Yes	7
	Male	86	MCL	560	–	5.1	3.5	180	Yes	4
	Female	73	MCL	560	–	8.2	3.9	260	Yes	1
	Male	72	WM	420	–	6.0	3.7	260	No	0
Median (±IQR)		73 (64–83)		–	–	5.7 (4.2–7.5)	3.7 (3.5–4.1)	210 (170–260)	2* (0.25–3.75)	

Abbreviations: ASA, acetylsalicylic acid; CLL, chronic lymphocytic leukaemia; CLOP: clopidogrel; DLBCL, diffuse large B‐cell lymphoma; IQR, interquartile range; ISTH‐BAT, International Society on Thrombosis and Haemostasis bleeding assessment tool; MCL, mantle cell lymphoma; MPV, mean platelet volume; PLT, platelet count; RBC, red blood cell count; WBC, white blood cell count; WM, Waldenstroms macroglobulinemia.

* *p* < 0.05, patients versus patients + ibrutinib.

**FIGURE 7:**
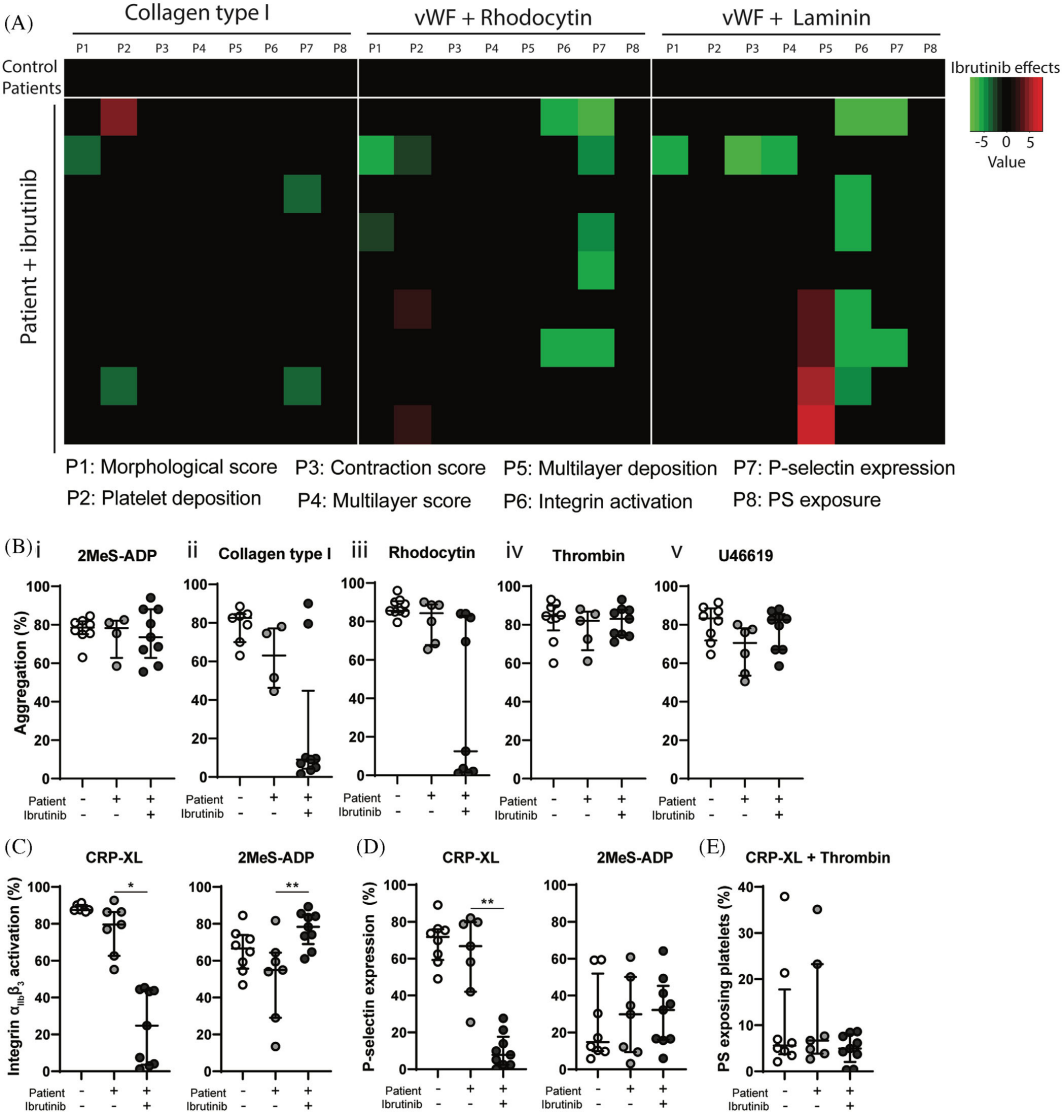
Ibrutinib inhibits whole blood thrombus formation under flow on multiple surfaces and GPVI‐induced platelet aggregation and activation responses in patients. Citrated whole blood was collected from healthy volunteers and 16 patients with(out) ibrutinib treatment. **(A)** Whole blood from healthy controls and patients was perfused for 3.5 min at a wall shear rate of 1000 s^–1^ over coated microspots containing collagen type I, vWF co‐coated with rhodocytin or vWF co‐coated with laminin. Subtraction heatmap representing the effect of ibrutinib treatment in individual patients. Parameters 1–8 are as described for Figure 2. Average values obtained with blood of patients without ibrutinib treatment was set at 0 for reference. Effects were filtered for changes greater than the 2x SD range of the different platelet parameters. **(B)** Light transmission aggregometry was induced in isolated platelets (250 × 10^9^ /L) by collagen (1 μg/ml), rhodocytin (1 μg/ml), 2MeS‐ADP (1 μM), U46619 (1 μM) or thrombin (1 nM). Scatter plots show the percentage of aggregation of healthy controls (white circles), patients (grey circles) or patients receiving ibrutinib (black circles). Each circle represents one individual. **(C and D)** Washed platelets (100 × 10^9^ /L) were stimulated with collagen‐related peptide (CRP‐XL, 5 μg/ml) or 2MeS‐ADP (1 μM) for 15 min and analysed by flow cytometry. Shown are percentages of platelets binding FITC‐labelled PAC1 mAb against integrin α_IIb_β_3_ **(C)** and AF647‐labelled CD62P mAb for *α*‐granule secretion **(D)**, respectively. **(E)** PS‐exposure was stimulated using both CRP‐XL (5 μg/ml) and thrombin (4 nM) for 60 min at 37°C. Scatter plots show the percentage of platelets binding FITC‐labelled Annexin A5. Data are represented as median ± interquartile range (*n* = 4‐9), *p < 0.05, **p < 0.01

Whole blood from patients was perfused over collagen type I or vWF plus rhodocytin or laminin. No differences in thrombus formation were observed between patients without ibrutinib and healthy controls (Figure S7). On collagen, treatment with ibrutinib reduced thrombus contraction and height (multilayer) compared to non‐treated patients. Furthermore, α_IIb_β_3_ activation_,_ P‐selectin expression and PS‐exposure were significantly reduced (Figures 7A and S7A). For thrombi formed on vWF plus rhodocytin or laminin, α_IIb_β_3_ activation and α‐granule secretion were reduced upon ibrutinib treatment (Figures 7A, S7B and S7C).

Aggregation of isolated platelets from ibrutinib‐treated patients showed on average no significant inhibition upon stimulation with any agonist (Figure 7B). However, platelets from five ibrutinib‐treated patients were highly nonresponsive to collagen stimulation, while platelets from two patients responded normally to collagen. Also, with rhodocytin, platelets from four patients receiving ibrutinib did not aggregate, while platelets from four patients showed normal aggregation. When platelet activation was examined by flow cytometry, α_IIb_β_3_ activation and P‐selectin expression were inhibited in CRP‐XL‐stimulated platelets from all ibrutinib‐treated patients (Figure 7C). Upon ADP stimulation, platelets from patients on treatment showed a slight increase in α_IIb_β_3_ activation, while no effects on secretion were observed (Figure 7C). PS‐exposure by thrombin plus CRP‐XL, was unchanged for all patients’ platelets (Figure 7D).

As 66% of our patients reported bleeding symptoms, we compared patients with significant bleeding (>2 ISTH‐BAT score) with patients with no/mild bleeding (0‐1 ISTH‐BAT score). Generally, no significant changes were observed in platelet aggregation and thrombus formation in patients with or without bleeding (Figure S8).

In summary, platelets from patients on ibrutinib treatment displayed inhibition of activation by GPVI as well as reduced adhesion and thrombus formation under flow over collagen and surfaces co‐coated with vWF. Furthermore, variable responses were seen with CLEC‐2 stimulation.

## 4 DISCUSSION

Due to their promising effects on progression‐free survival, Btk inhibitors are increasingly prescribed in haematological malignancies [4, 5]. Btk inhibitors require lifelong treatment, and the high percentage of discontinuation due to side effects (including bleeding tendency) stresses the importance of understanding and managing these [6, 7, 8]. We found that ibrutinib and acalabrutinib inhibit both GPVI‐ and CLEC‐2‐mediated platelet aggregation in a dose‐dependent manner. Also, all inhibitors dose‐dependently impaired GPVI‐induced α_IIb_β_3_ activation and *α*‐granule secretion. Ibrutinib reduced thrombus formation on surfaces co‐coated with vWF, but not on collagen. Moreover, we are the first to study the effects on platelet function of MK‐1026, a reversible Btk inhibitor.

Other studies investigating effects of Btk inhibitors on platelet function have described different findings. Several papers have shown inhibition of the GPVI pathway by ibrutinib or acalabrutinib, either directly via Btk or due to off‐target effects on Src kinases or other downstream proteins [11, 12, 23, 27, 28, 29, 30, 31]. Others have implicated that GPIb‐ [29, 32] or α_IIb_β_3_‐mediated signalling [28, 31] were affected by ibrutinib. In agreement, in the present study ibrutinib also inhibited thrombus formation on ristocetin and fibrinogen co‐coated with vWF. Levade et al have also shown that ibrutinib affected platelet adhesion to vWF only under flow but did not assess acalabrutinib [22]. Furthermore, we found that both ibrutinib and acalabrutinib impaired GPVI‐mediated aggregation but not platelet adhesion on collagen under flow. Previous studies found conflicting results with regard to the effects of ibrutinib on collagen adhesion, with some who did find inhibition [12, 33] and others that could not [34]. A possible explanation for this is that platelet adhesion to collagen is also regulated by integrin α_2_β_1_, which does not signal via Btk [34]. Our data now directly compare the differences between ibrutinib and acalabrutinib in whole blood thrombus formation under flow on six different surfaces and show for the first time that ibrutinib, but not acalabrutinib, impairs platelet adhesion surfaces co‐coated with vWF. This might play a role in the increased incidence of major bleeding seen in ibrutinib treatment compared to acalabrutinib.

Although GPVI is important for platelet adhesion and activation on collagen, recent studies have shown that GPVI inhibitors do not impair haemostasis [16]. GPVI‐deficient patients exhibit only a mild bleeding diathesis [17], and GPVI depletion in mice did not increase bleeding tendency [19, 20]. Therefore, the bleeding tendency seen with Btk inhibitors cannot solely be mediated by GPVI. Depletion of both GPVI and CLEC‐2 from mouse platelets did impair haemostasis [21]. In line with this, we found that Btk inhibitors inhibited both GPVI‐ and CLEC‐2‐mediated aggregation.

Only few studies have investigated Btk‐KO mice or X‐linked agammaglobulinemia (XLA) patients, a Btk deficiency in humans. Btk‐KO mice showed impaired GPVI, CLEC‐2 and GPIb signalling [35, 36, 37, 38]. In line with this, we found decreased GPVI‐induced platelet granule secretion and thrombus formation in Btk‐KO mice. XLA patients demonstrated impaired GPVI‐ or CLEC‐2‐induced PLCγ2 phosphorylation and platelet aggregation [39, 40]. As compared to XLA patients, ibrutinib has similar effects on GPVI, CLEC‐2 and GPIb signalling. However, XLA patients are not associated with an increased bleeding risk [12]. This suggests that Btk inhibitors affect other targets, most notably Tec, which can substitute for Btk [35] and is also inhibited by ibrutinib and acalabrutinib [12]. Also, off‐target effects on Src kinases have been implicated in the bleeding tendency [11, 12]. We assessed off‐target effects on GPVI‐induced tyrosine kinase phosphorylation in platelets by using the PamGene assay. These results showed that ibrutinib and acalabrutinib showed the same off‐target effects, although the effects of acalabrutinib were less strong. Importantly both inhibitors had an off‐target effect on Tec and Src kinases, questioning the previous conclusions that these were responsible for the increased severe bleeding seen with ibrutinib compared to acalabrutinib.

When comparing results found in vitro in human and mice, we found that in human samples, Btk inhibitors did not alter collagen‐mediated adhesion under flow in agreement with Zheng et al [31], whereas in mice, Btk inhibition (either by KO mice or acalabrutinib) reduced this response. Btk‐dependent signalling is completely absent in KO mice, and therefore, this may result in stronger effects as compared to pharmacological inhibition. Btk proteins display 99.4% similarity between human and mice [41], so a species‐dependent interaction with an inhibitor cannot explain the observed differences. A possibility could be different bioavailability between mouse and man. Ibrutinib and acalabrutinib are highly plasma protein‐bound in human blood, 97.3% and 97.5%, respectively [42, 43], while acalabrutinib was reported to have lower protein binding in mouse blood (75.4%) [43]. This may contribute to the more pronounced effects of acalabrutinib in mouse blood as compared to human blood. We also observed different results between human and mouse platelets with MK‐1026 (same concentration) in flow cytometric experiments. This may be explained by the use of murine whole blood in these experiments. Mouse blood contains ∼three times more platelets as compared to human blood, and plasma proteins are also likely to bind MK‐1026. Altogether, this may explain the absent effect of MK‐1026 on platelet functions of wild‐type mice.

As our data propose a direct role for Btk in impaired platelet function, we tested the reversible Btk inhibitor MK‐1026. MK‐1026 showed promising effects on ibrutinib resistant CLL cells in vitro and is currently being tested in clinical trials [14]. In the PamGene kinase assay MK‐1026 appeared to have less off‐target effects in platelets upon GPVI stimulation, in combination with a less strong inhibition profile as compared to the irreversible inhibitors. MK‐1026 reduced GPVI‐ and CLEC‐2‐mediated aggregation, as well as GPVI‐induced platelet activation in washed platelets, largely similar to acalabrutinib. In human and mouse blood, MK‐1026 did not impair collagen‐induced thrombus formation under flow, in contrast to acalabrutinib which did affect this response in mouse blood. Hence, this reversible inhibitor had less inhibitory effects on platelets as compared to ibrutinib, in agreement with a recent study [31]. Hence, MK‐1026 could be expected to show a slightly reduced or comparable bleeding tendency compared to acalabrutinib.

The present dose‐response experiments, in line with previous studies [11, 33], show that at similar dose, ibrutinib is a more effective platelet inhibitor compared to other Btk inhibitors. This has been attributed to the inhibition of drug efflux pumps by ibrutinib [33]. We observed that higher concentrations of inhibitors are required to inhibit platelet aggregation in the presence of plasma, as compared to washed platelets. This in line with previous observations with other TKIs [44, 45]. This is most likely caused by binding of ibrutinib and acalabrutinib to albumin [46, 47, 48]. It has been reported that prolongation of the incubation time lowered the IC_50_ values of ibrutinib and acalabrutinib for GPVI‐mediated aggregation [49]. However, in that study a much lower collagen concentration was used, which may be more sensitive to longer incubation with low doses of inhibitors. Furthermore, that study did not include CLEC‐2‐dependent platelet responses [49]. Although inhibitor concentration could play a role in the extent of inhibition, we showed that ibrutinib and acalabrutinib influence additional platelet pathways as compared to MK‐1026, suggesting that the extent of Btk inhibition might not be the reason for the increased bleeding tendency.

Previous studies assessing which platelet pathways are involved in bleeding tendency in patients using ibrutinib found conflicting results. Some showed a correlation with collagen‐induced aggregation [12, 22, 50] and some with platelet adhesion to collagen [12], whereas others could not find this [22]. Bleeding tendency was also associated with adhesion to vWF under flow [22], and one study found a correlation with ristocetin‐induced platelet aggregation [32], which another study could not replicate [28]. Ibrutinib can also induce shedding of GPIbα, GPIX and integrin α_IIb_β_3_ in patients, but the correlation with bleeding remains unknown [51].

A recent study assessed platelet parameters in patients with CLL and mantle cell lymphoma (MCL) [52], showing correlations between bleeding tendency, thrombocytopenia and decreased ADP‐induced platelet aggregation. However, a drawback of this study was that a large patient proportion was thrombocytopenic, which can directly influence platelet aggregation, as platelet concentration in PRP was not reported to be adjusted. This impaired the establishment if ibrutinib or platelet count affected aggregation response. In our patients with (in general) normal platelet counts, we also observed significant differences in platelet responses to several stimuli of patients using ibrutinib. Furthermore, 66% of the included patients with ibrutinib treatment reported bleeding symptoms. Although we could show that in patients, similar to healthy volunteers, ibrutinib inhibited GPVI signalling, with variable effects on CLEC‐2, as well as reduced thrombus formation to surfaces co‐coated with vWF, this could not differentiate for the bleeding tendency. Generally, the patients showed a large variation in measurement outcomes, which might be influenced by clinical factors. However, with patients on ibrutinib treatment, we have directly compared whole blood thrombus formation under flow on multiple surfaces, including vWF plus rhodocytin and laminin, which has not been reported with ibrutinib‐treated patients thus far.

In conclusion, the present work demonstrated that ibrutinib, acalabrutinib and MK‐1026 inhibited GPVI‐ and CLEC‐2‐mediated platelet aggregation, but only ibrutinib also inhibited GPVI‐induced platelet activation and thrombus formation on surfaces co‐coated with vWF. The novel reversible BTK inhibitor MK‐1026 might therefore be promising for future treatment in patients at risk for bleeding.

## AUTHOR CONTRIBUTIONS

Bibian M.E. Tullemans, Mieke F.A. Karel, Valentine Léopold, Marieke S. ten Brink, Constance C.F.M.J. Baaten, Sanne L. Maas and Emiel P.C. van der Vorst acquired, analysed and interpreted data. Alex F. de Vos provided Btk‐deficient mice. Johannes A. Eble contributed essential tools. Marten R. Nijziel and Theodora A.M. Claushuis provided ethical approval and included patients. Judith M.E.M. Cosemans and Johan W.M. Heemskerk provided laboratory space with equipment and supervision. Theodora A.M. Claushuis and Marijke J.E. Kuijpers designed and supervised the research and interpreted data. Bibian M.E. Tullemans, Theodora A.M. Claushuis and Marijke J.E. Kuijpers wrote and revised the paper. All the authors provided critical feedback, edited and approved the final manuscript.

## Supporting information

Figure S1

Figure S2

Figure S3

Figure S4

Figure S5

Figure S6

Figure S7

Figure S8

Supporting Information

Table S1

## Data Availability

The data that support the findings of this study are available from the corresponding author upon reasonable request.
