# Supplementary figures and images for: Comparison of inhibitory effects of irreversible and reversible Btk inhibitors on platelet function
Source: EJHaem. 2021 Aug 10;2(4):685–99. doi: 10.1002/jha2.269 (PMC9175945; doi:10.1002/jha2.269)

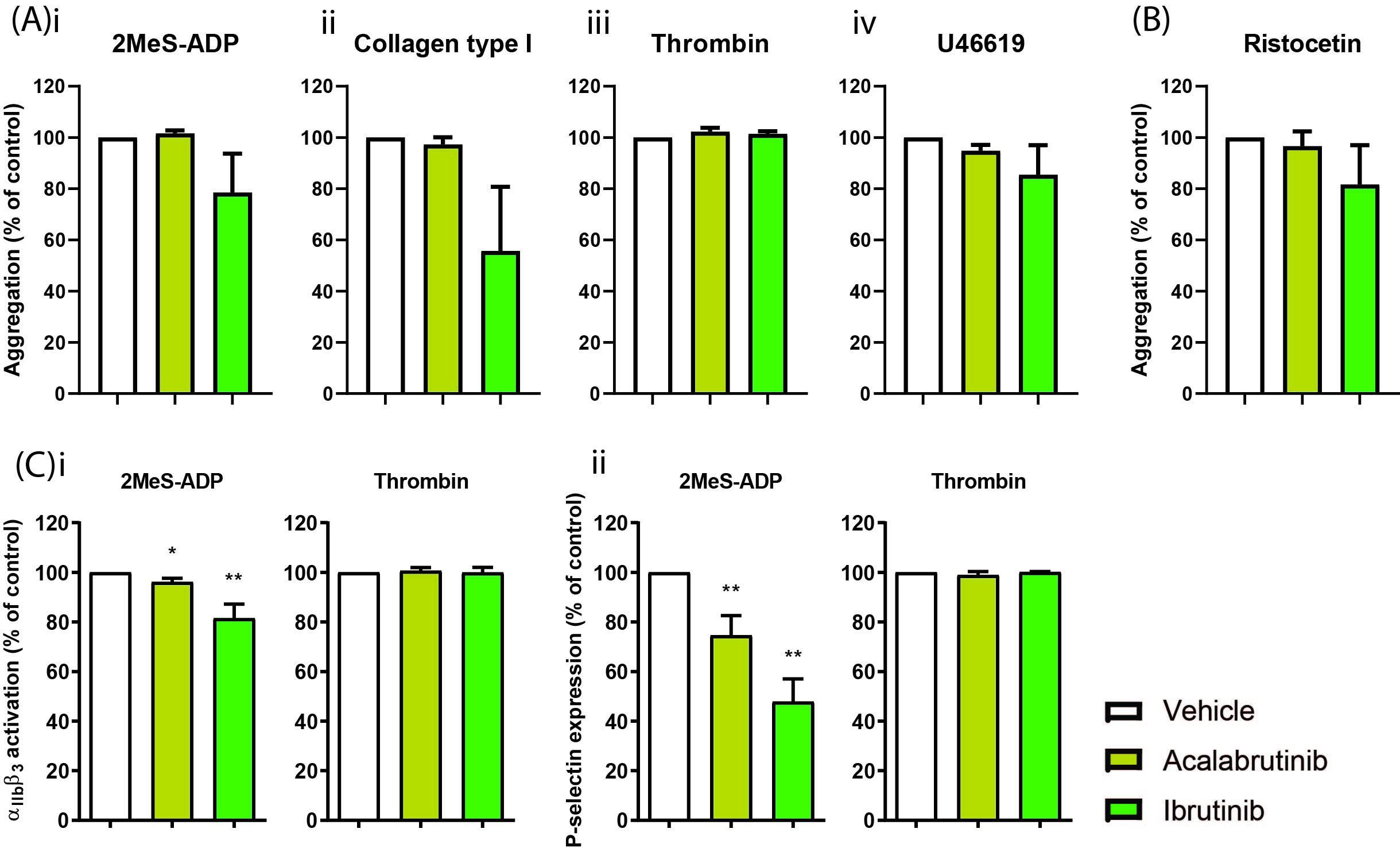

Supplement: Supplementary file 1 — Figure S1 [file JHA2-2-685-s005.jpg]

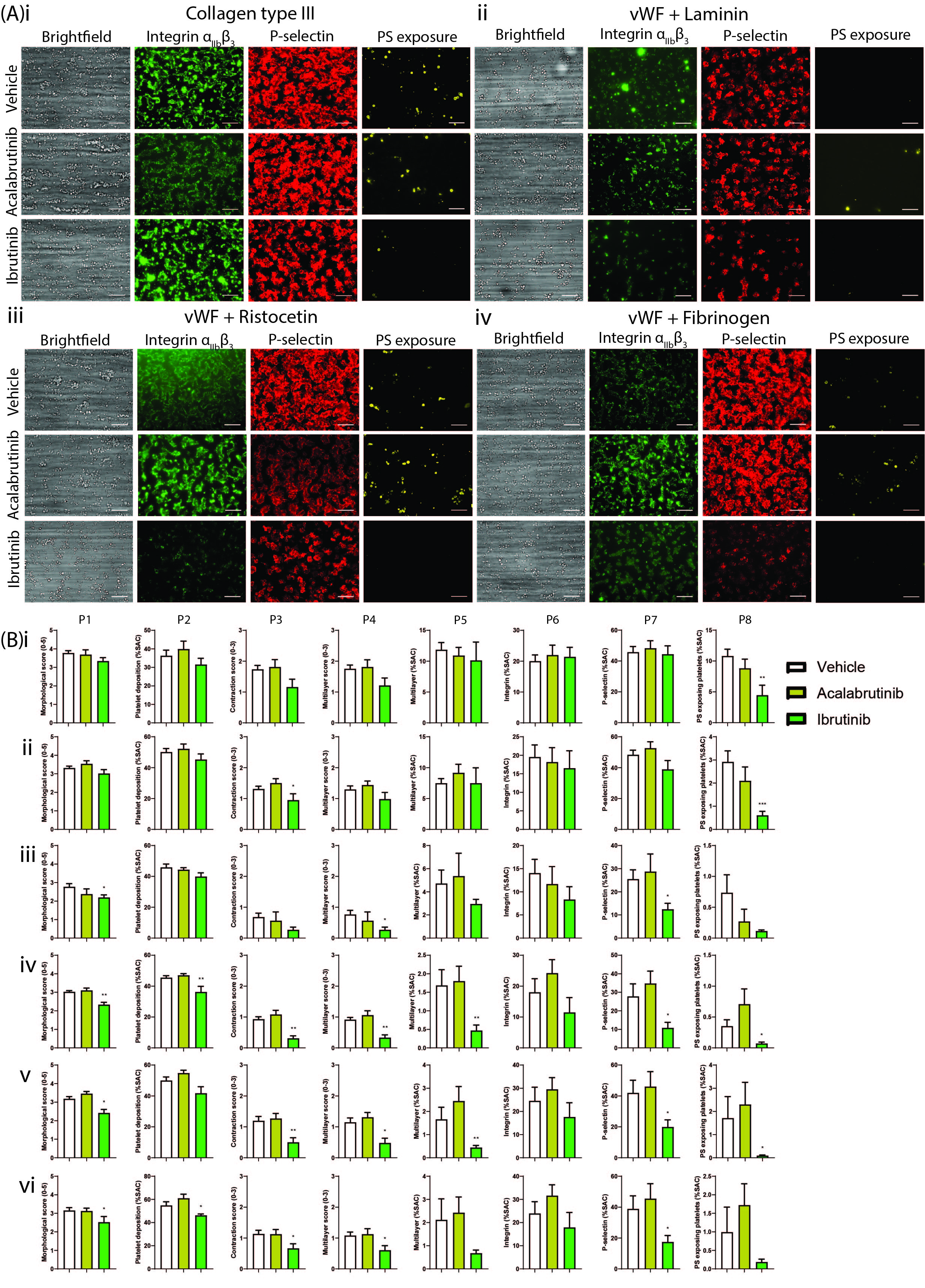

Supplement: Supplementary file 2 — Figure S2 [file JHA2-2-685-s010.jpg]

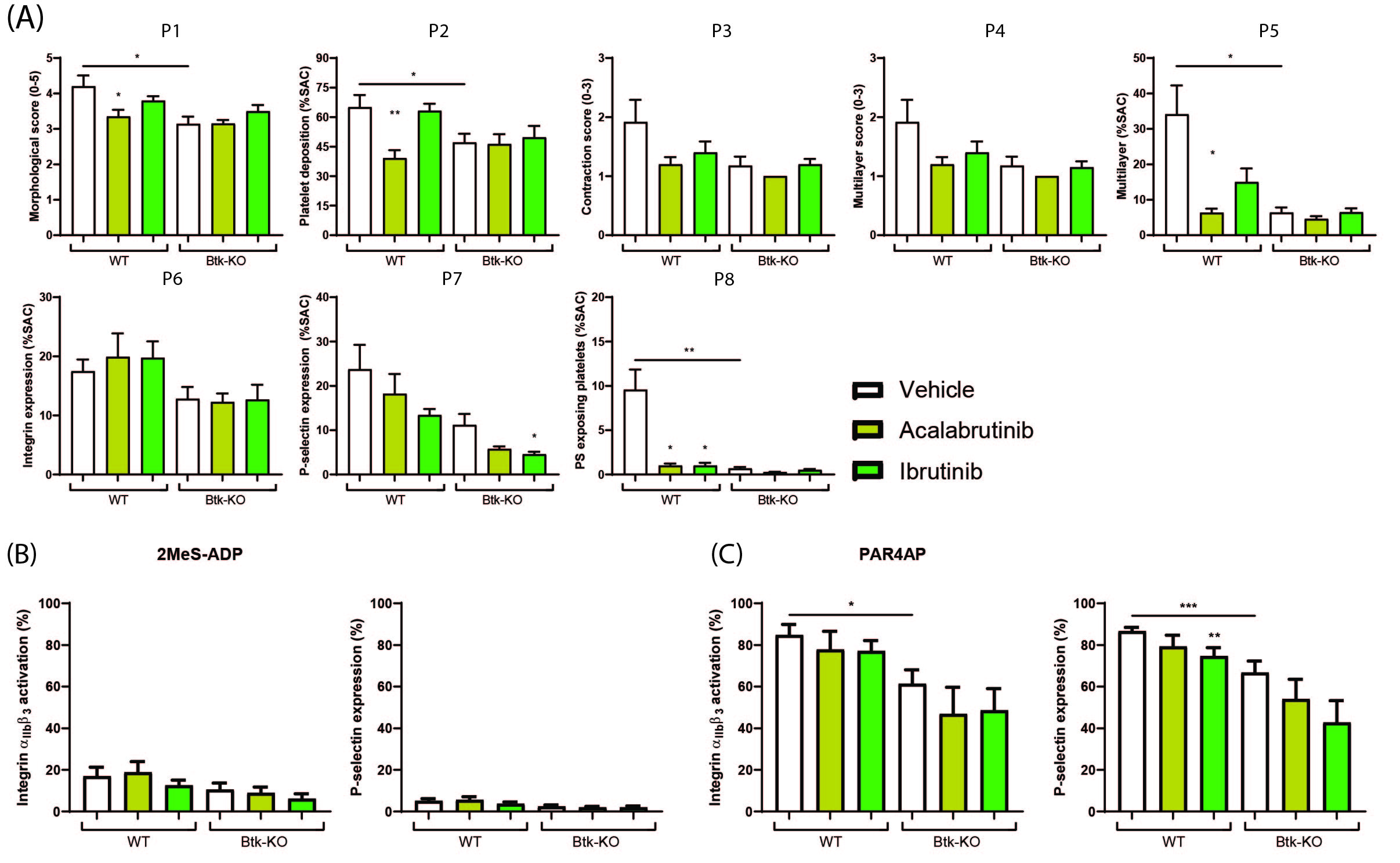

Supplement: Supplementary file 3 — Figure S3 [file JHA2-2-685-s002.jpg]

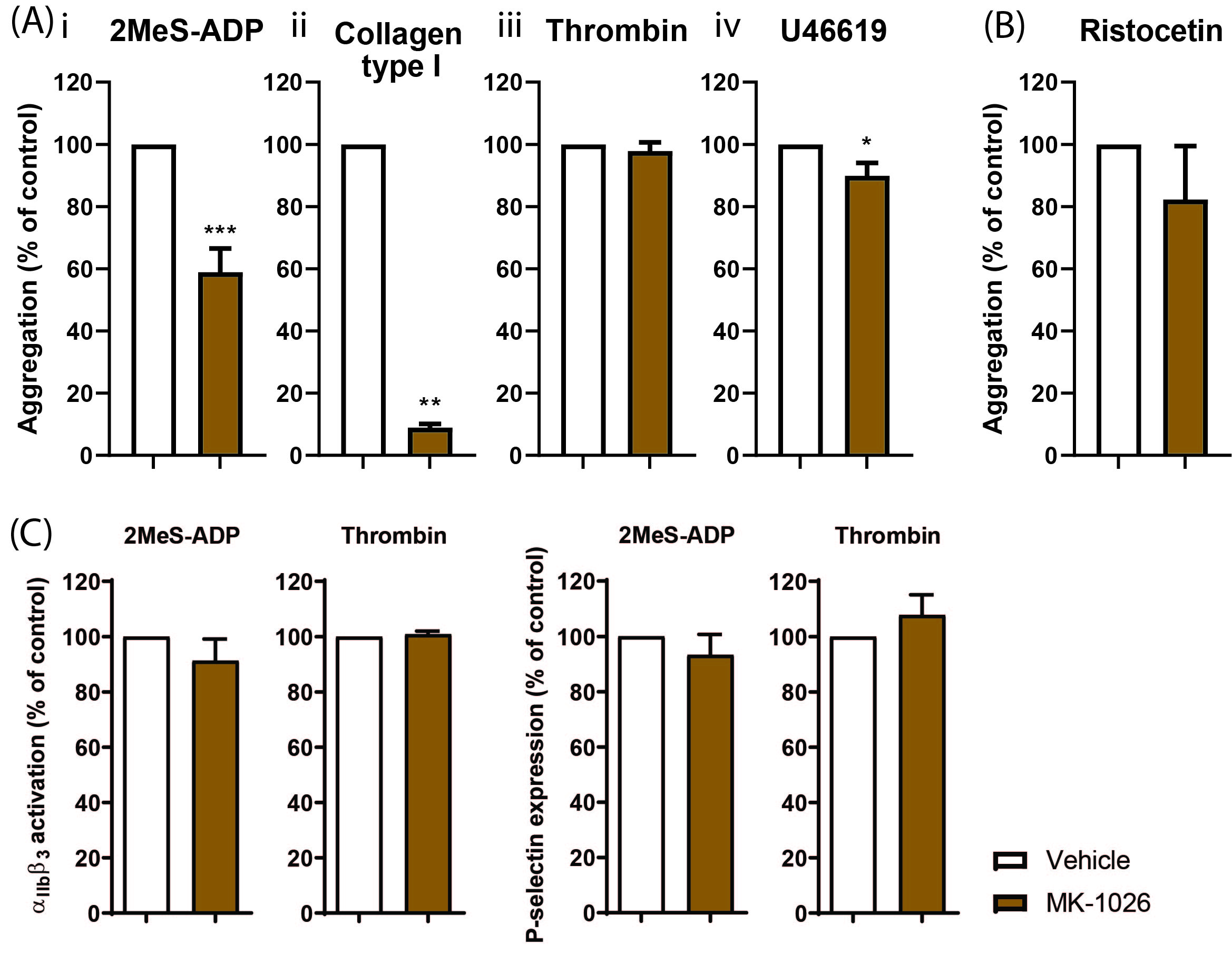

Supplement: Supplementary file 4 — Figure S4 [file JHA2-2-685-s001.jpg]

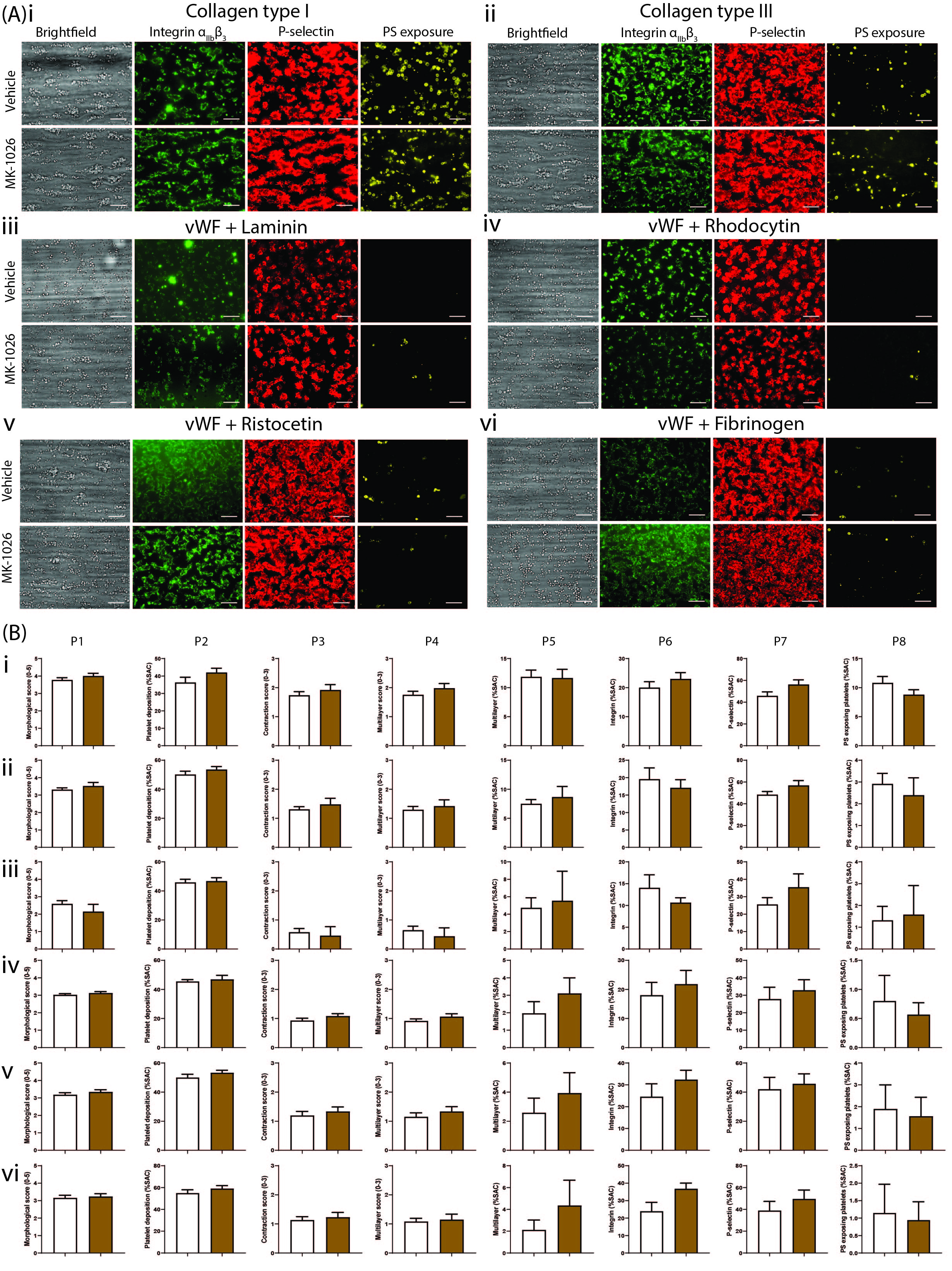

Supplement: Supplementary file 5 — Figure S5 [file JHA2-2-685-s006.jpg]

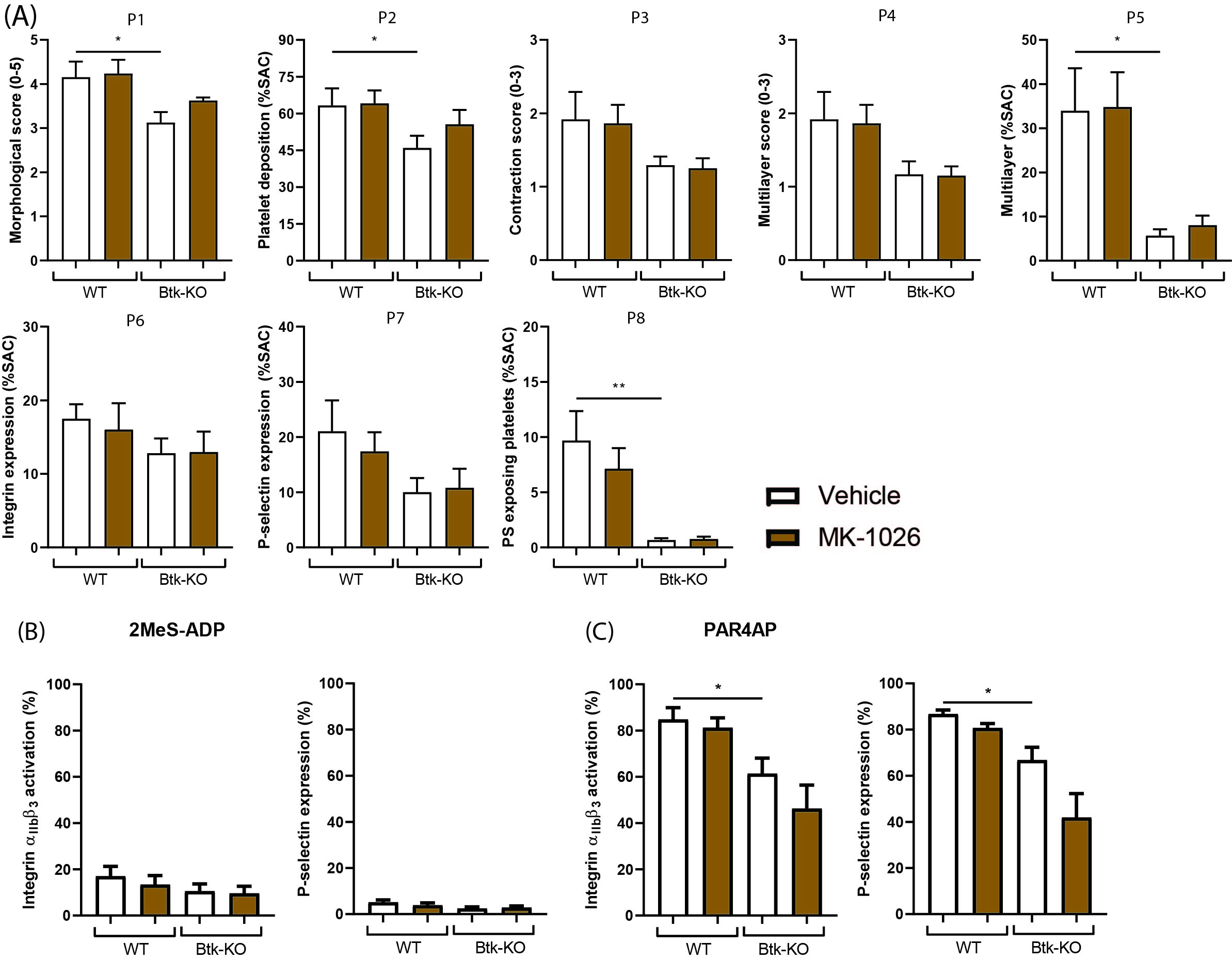

Supplement: Supplementary file 6 — Figure S6 [file JHA2-2-685-s008.jpg]

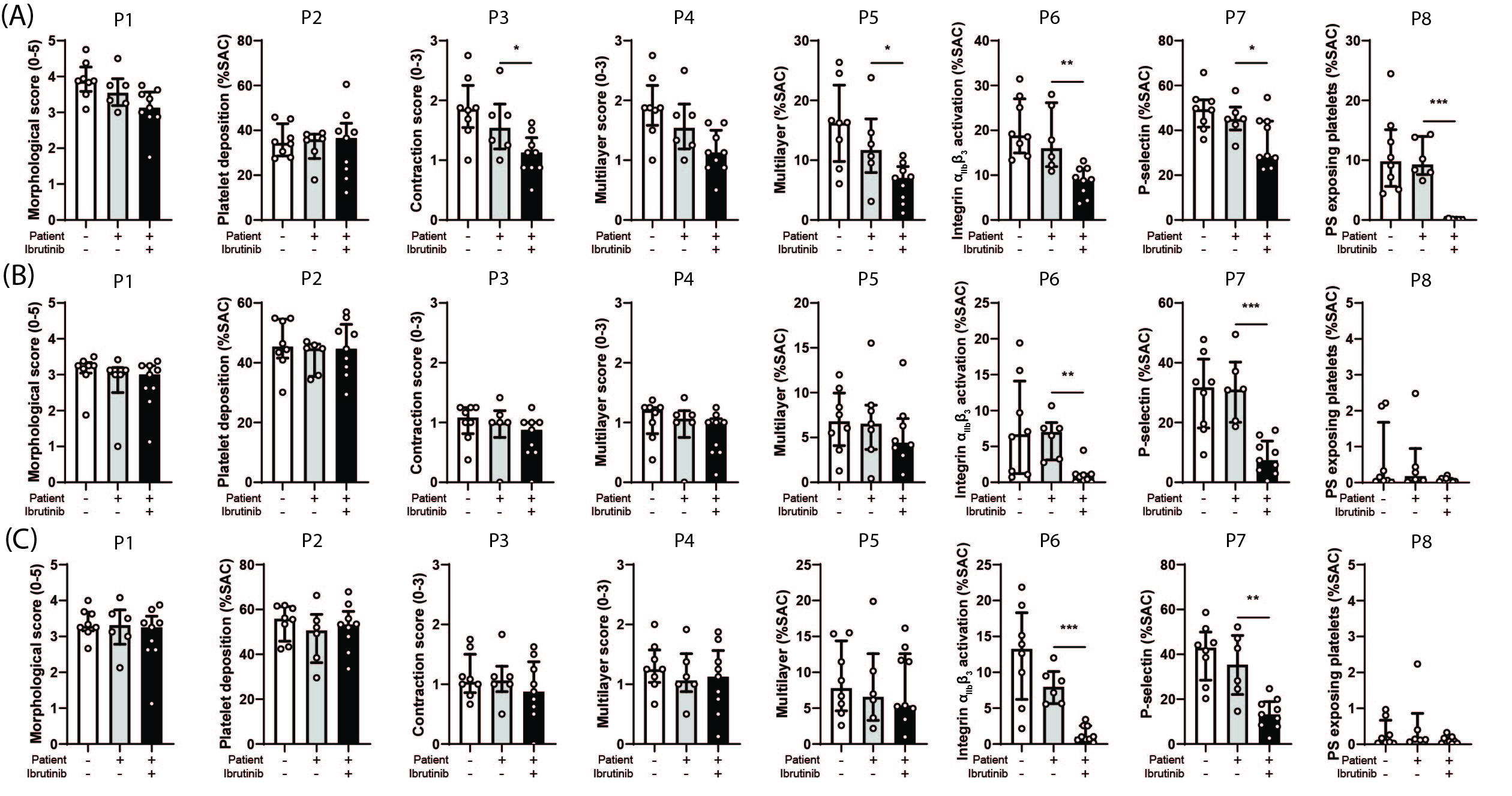

Supplement: Supplementary file 7 — Figure S7 [file JHA2-2-685-s009.jpg]

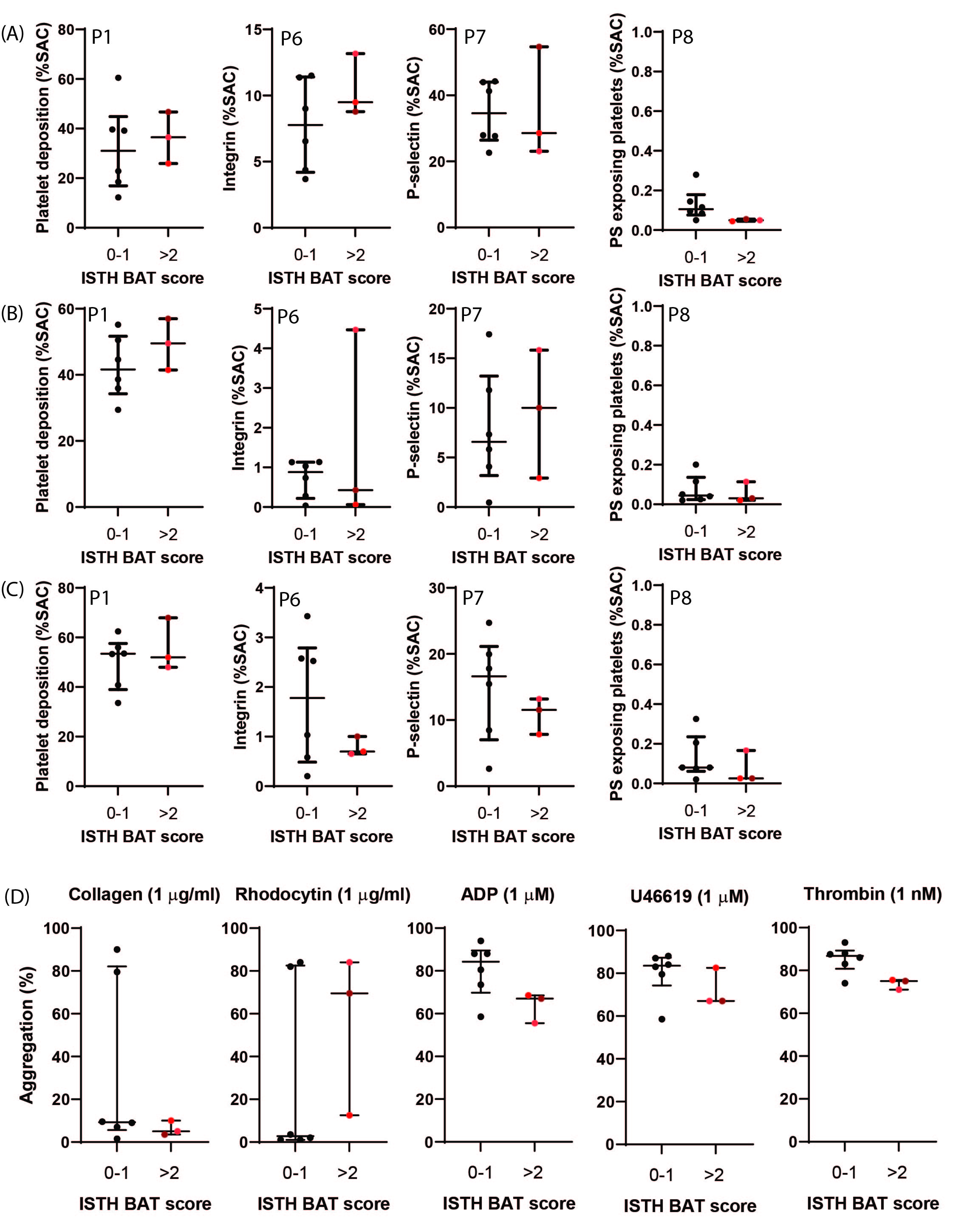

Supplement: Supplementary file 8 — Figure S8 [file JHA2-2-685-s003.jpg]
