# Supplementary material for: Comparison of inhibitory effects of irreversible and reversible Btk inhibitors on platelet function
Source: EJHaem. 2021 Aug 10;2(4):685–99. doi: 10.1002/jha2.269 (PMC9175945; doi:10.1002/jha2.269)
Supplement: Supplementary file 9 — Supporting Information [file JHA2-2-685-s004.docx]

**Comparison of inhibitory effects of irreversible and reversible Btk inhibitors on platelet function**

Bibian M.E. Tullemans et al.

**Supplemental Material and Methods**

***Materials***

Acalabrutinib was obtained from Selleckchem (Houston, TX, USA), MK-1026 (formerly ARQ-531) was a provided by ArQule, Inc., a wholly-owned subsidiary of Merck & Co. (Kenilworth NJ, USA) and ibrutinib was purchased from Toronto Research Chemicals (North York, Canada). Bovine serum albumin (BSA), D(+)-glucose, unfractionated heparin and apyrase were purchased from Sigma-Aldrich (Saint Louis MO, USA). Low molecular weight heparin (Fragmin®) was from Prizer (Capelle a/d IJssel, The Netherlands). Horm® collagen type I came from Takeda (Hoofddorp, The Netherlands), whereas the agonists collagen-related peptide crosslinked (CRP-XL) and von Willebrand factor III (vWF-III) were obtained from CambCol Laboratories (Cambridge, UK). Thrombin was from Enzyme Research Laboratories Inc (South Bend, IN, USA) and protease-activated receptor 4 activating peptide (PAR4-AP) was obtained from Bachem (Bubendorf, Switzerland). U46619 (thromboxane A_2_ receptor agonist) came from Cayman Chemicals (Ann Arbor MI, USA). 2-Methylthio-adenosine-diphosphate (2MeS-ADP) and D-phenylalanyl-prolyl-arginyl chloromethyl ketone (PPACK) were obtained from Santa Cruz Biotechnology (Dallas TX, USA). Laminin was purchased from Octapharma (Lachen, Switzerland), whereas rhodocytin was purified from *Calloselasma rhodostoma* venom as described previously.^1^ Fura-2-AM was obtained from Invitrogen (Carlsbad CA, USA). Fluorescein isothiocyanate (FITC)-labelled PAC1 monoclonal antibody (mAb) against activated human integrin α_IIb_β_3_ were purchased from BD Bioscience (nr. 340507; Franklin Lakes NJ, USA). FITC-conjugated α-fibrinogen mAb was purchased from DAKO (F0111; Santa Clara CA, USA). Alexa Fluor (AF)568 and AF647-conjugated annexin A5 were purchased from Molecular Probes, Life Technologies (New York NY, USA). AF647-labelled CD62-P mAb was obtained from Biolegend (London, UK). FITC-conjugated annexin A5 was from Pharmatarget (Maastricht, The Netherlands), whereas FITC-conjugated lactadherin came from Haematologic Technologies (Essex Junction VT, USA). FITC-labelled rat anti-mouse CD62P mAb and phycoerythrin (PE) labelled rat anti-mouse JON/A mAb were obtained from Emfret Analytics (Würzburg, Germany).

***Blood collection and platelet isolation from healthy volunteers and patients***

With approval from the medical ethics committee from the Maastricht University Medical Centre+ (MUMC+), blood was collected from healthy volunteers. Furthermore, blood was collected from patients diagnosed with chronic lymphocytic leukaemia (CLL), mantle cell lymphoma and Waldenstroms macroglobulinemia with or without ibrutinib treatment at the Catharina Hospital in Eindhoven (the Netherlands) with approval of the local medical ethical committee. This study was performed in accordance with the declaration of Helsinki and informed consent was obtained from all participants. Blood was collected in 3.2% trisodium citrate tubes by venipuncture, after discarding the first 3 mL of blood. Blood cell counts and haematological parameters were assessed of all blood samples using a Sysmex XP300 (Kobe, Japan). Patients characteristic and ISTH-BAT score^2^ were acquired for each patient.

Platelet-rich plasma (PRP) or platelets were isolated from whole blood as described previously ^3,4^. Washed platelets were resuspended in Hepes buffer, pH 7.45 (10 mM Hepes, 136 mM NaCl, 2.7 mM KCl, 2 mM MgCl_2_, 1 mg/mL glucose, and 1 mg/ml BSA). Platelet concentrations were adjusted as stated per assay.

Blood collection from animals

Specific-pathogen-free Btk-KO mice^5^ and littermate controls (wild-types, WT) were housed in the Animal Research Institute Amsterdam facility under standard care. Studies were reviewed and approved by the Central Authority for Scientific Procedures on Animals (CCD) and the Animal Welfare Body (IvD) of the Academic Medical Centre, University of Amsterdam (ID number: 17-4125-1-80). The animal care and use protocol adhered to the Dutch Experiments on Animals Act (WOD) and European Directive of 22 September 2010 (Directive 2010/63/EU) in addition to the Directive of 6 May 2009 (Directive 2009/41/EC).

Only male mice between 8 and 12 weeks of age were used for experiments. Citrated blood was drawn from WT and Btk-KO mice from the vena cava inferior under full anaesthesia induced by intraperitoneal injection of ketamine (125 mg/kg bodyweight) and dexmedetomidine (300 µg/kg bodyweight). After blood collection mice were sacrificed by severing the diaphragm.

Whole blood thrombus formation under flow

Thrombus formation under flow with human blood was performed as described ^6^. Briefly, glass coverslips were coated with 3 microspots of collagen type I (100 µg/mL), collagen type III (100 µg/mL), vWF (50 µg/mL) co-coated with laminin (100 µg/mL), vWF co-coated with rhodocytin (250 µg/mL), vWF co-coated with ristocetin (250 µg/mL), or vWF co-coated with fibrinogen (250 µg/mL), and mounted in a parallel plate flow chamber. Citrated blood samples from healthy donors were incubated with vehicle or 5 µM of Btk inhibitor (acalabrutinib, ibrutinib or MK-1026) for 10 minutes at room temperature. Blood samples from CLL patients were used directly. Whole blood from healthy volunteers or CLL patients was recalcified in the presence of PPACK (40 µM), and perfused at a wall shear rate of 1000 s^-1^ for 3.5 minutes. Platelet activation properties were determined by post-perfusion with Hepes buffer (with additional 2 mM CaCl_2_ and 1 U/mL heparin) containing FITC-conjugated α-fibrinogen mAb (1:80), AF647-conjugated CD62-P (1:100), and AF568-conjugated annexin A5 (1:200).

Thrombus formation under flow with mouse blood was performed as described.^7^ Glass coverslips were coated with collagen type I (100 µg/mL) and blood samples, in the presence of PPACK (57.1 µM) and Fragmin (35.7 µM), were incubated with 5 µM Btk inhibitor (acalabrutinib, ibrutinib or MK-1026) for 10 minutes at 37°C. Next, the blood samples were recalcified and perfused at a shear rate of 1000 s^−1^ for 3,5 minutes. To measure platelet activation status, thrombi were post-stained with Tyrode Hepes buffer, pH 7.45 (5 mM Hepes, 136 mM NaCl, 2.7 mM KCl, 0.42 mM NaH_2_PO_4_, 1 mg/mL glucose, 1 mg/ml BSA, 2 mM CaCl_2_, 2 mM MgCl­_2_ and 1 U/mL heparin) containing PE-labelled Jon-A (1:20) and FITC-conjugated anti-P-selectin mAb (1:40) and AF647-labelled Annexin-A5 (1:200).

For both human and mouse experiments, brightfield and fluorescent images were captured using an EVOS microscope (Bothel, WA, USA). Images were analysed using specific scripts in the open-access Fiji software (Laboratory for Optical and Computational Instrumentation, University of Wisconsin-Madison, WI, USA) as described ^6,8^. Values for the following parameters of thrombus formation and platelet activation were obtained: morphological score of platelet adhesion and thrombus formation (P1; scale 0-5), surface area coverage of adhered platelets (P2; %SAC), platelet aggregate contraction score (P3; scale 0-3), platelet aggregate multilayer score (P4; scale 0-3), coverage of multi-layered platelet aggregation (P5; %SAC), integrin α_IIb_β_3_ activation (P6; %SAC), P-selectin expression (P7; %SAC) and PS exposure (P8; %SAC). For comparative data analysis, cumulative histograms were generated of scaled values from 0-10 for each parameter. For patient data subtraction heatmap representing the effect of ibrutinib treatment in individual patients. Average values obtained with blood of patients without ibrutinib treatment was set at 0 for reference. Effects were filtered for changes greater than the 2x SD range of the different platelet parameters.

Light transmission aggregometry

Isolated platelets (250x10^9^ platelets/L) from healthy volunteers were incubated with vehicle or 3.3 µM of Btk inhibitor (acalabrutinib, ibrutinib or MK-1026) for 10 minutes at 37°C, whereas platelets from CLL patients were used directly after preheating to 37°C for 10 minutes. Aggregation response was induced by collagen type I (1 µg/mL), 2MeS-ADP (1 µM), U46619 (1 µM), rhodocytin (1 µg/mL) or thrombin (1 nM). Platelet aggregation was recorded using a Chronolog optical aggregometer (Havertown PA, USA) and maximum amplitude was quantified at 8 minutes after agonist addition.

Flow cytometry

Washed platelets (100x10^9^ platelets/L) from healthy volunteers or CLL patients were supplemented with 2 mM CaCl_2_. Samples from healthy volunteers were incubated with vehicle or 1 µM of Btk inhibitor (acalabrutinib, ibrutinib or MK-1026) for 10 minutes at room temperature, whereas patient samples were used directly. Platelets were stimulated by CRP-XL (5 μg/mL), 2MeS-ADP (1 μM), or thrombin (1 nM) for 15 minutes at RT. Activation of integrin αIIbβ3 and P-selectin expression were determined using FITC-conjugated PAC1 mAb (1:20) and AF647-conjugated CD62-P mAb (1:40) respectively. For measuring phosphatidyl-serine (PS) exposure platelets were activated with a combination of CRP-XL (5 μg/mL) and thrombin (4 nM) for 1 hour at 37°C and labelled with FITC-conjugated annexin A5. Flow cytometry was performed in duplicates using a BD Accuri C6^TM^ flow cytometer and accompanying software (Erembodegem, Belgium).

Platelet activation in whole blood from WT and Btk-KO mice was performed using pooled blood samples (5 μl), in the presence of PPACK (57.1 µM) and Fragmin (35.7 µM), incubated with 5 µM Btk inhibitor (acalabrutinib, ibrutinib or MK-1026) for 10 minutes at 37°C. Platelets were then stimulated for 30 minutes at room temperature with control buffer (PBS), CRP-XL (1 μg/ml), 2MeS-ADP (1 μM) or PAR4-AP (0.1 mg/ml). Platelet activation was assessed by flow cytometry using APC-conjugated anti-CD61, BB700-labelled anti-CD62P, PE-conjugated JON/A and FITC-labelled lactadherin. The labelling was terminated by addition of 40 volumes of buffer. Analysis was carried out immediately by flow cytometry using a CytoFLEX (Beckman Coulter, Brea, CA) until 10,000 CD61+ events were recorded. All stimulations and staining were done in duplicate. Compensation settings were validated using single-stained controls. Percentage of positivity was determined using unstimulated controls. Data analysis was done using FlowJo^TM^ v10 (BD Biosciences, San Jose, CA).

Cytosolic Ca^2+^ measurements

Washed platelets (200x10^9^ platelets/L) were loaded with Fura-2 acetoxymethyl ester and changes in cytosolic Ca^2+^ ([Ca^2+^]_i_) were measured in 96-well plates using a FlexStation 3 (Molecular Devices, San Jose, CA, USA) as described.^9,10^ In brief, platelet suspension was preincubated with vehicle or 1 µM Btk inhibitor (acalabrutinib, ibrutinib or MK-1026) for 10 minutes at RT. Fura-2 loaded platelets in presence of CaCl_2_ were stimulated by CRP-XL (10 µg/mL). Changes in Fura-2 fluorescence were measured in duplicate and ratio values were calculated and are presented as [Ca^2+^]_i_ in nM.

***PamGene kinase assay***

Washed platelets (500 x 10^9^/L) were pretreated with vehicle (control) or 1 μM ibrutinib, acalabrutinib or MK-1026 for 10 minutes at 37°C and were subsequently stimulated with 5 μg/mL CRP-XL in the presence of 2 mM CaCl_2_. Unstimulated, resting platelets served as control. After 90 seconds of stimulation, samples were lysed by adding 1:1 M-PER Mammalian Extraction Buffer containing Halt Phosphatase Inhibitor and EDTA-free Halt Protease Inhibitor Cocktail (1:100 each; Thermo Fischer Scientific). Samples were lysed for 15 minutes on ice and afterwards centrifuged for 15 minutes at 10,000g at 4°C. Supernatants were collected and protein content was quantified with a BioRad DC protein kit (Hercules CA, USA).

Tyrosine kinase profiles were determined using the PamChip® peptide tyrosine kinase microarray system on PamStation®12 (PTK; PamGene International, ´s-Hertogenbosch, The Netherlands). Each PTK-PamChip® array contains 144 individual phospho-site(s) that are peptide sequences derived from substrates for TKs. Each peptide on the chip builds a 15-amino acid sequence representing a putative endogenous phosphorylation site which functions as a TK substrate. The phosphorylation of the peptides is visualized by detection of the fluorescent signal which is emitted as a result of the binding of the FITC-conjugated PY20 anti-phosphotyrosine antibody.

For the PTK assay, 7.5 µg of protein was applied per array (N=4 per condition) and carried out using the standard protocol supplied by Pamgene. All reagents used for PTK activity profiling were supplied by Pamgene International B.V. Initially, to prepare the PTK Basic Mix, the freshly frozen lysate was added to 4 µL of 10 x protein PTK reaction buffer (PK), 0.4 µL of 100 x bovine serum albumin (BSA), 0.4 µL of 1 M dithiothreitol (DTT) solution, 4 µL of 10 x PTK additive, 4 µL of 4 mM ATP and 0.6 µL of monoclonal anti-phosphotyrosine FITC-conjugate detection antibody (clone PY20). Total volume of the PTK Basic Mix was adjusted to 40 µL by adding distilled water (H20). Before loading the PTK Basic Mix on the array, a blocking step was performed applying 30 µL of 2% BSA to the middle of every array and washing with PTK solution for PamChip® preprocessing. Next, 40 µL of PTK Basic Mix were applied to each array of the PamChips®. Then, the microarray assays were run for 94 cycles. An image was recorded by a CCD camera PamStation®12 at kinetic read cycles 32–93 at 10, 50 and 200 ms and at end-level read cycle at 10, 20, 50, 100 and 200 ms. The spot intensity at each time point was quantified (and corrected for local background) using the BioNavigator software version 6.3 (PamGene International, 's-Hertogenbosch, The Netherlands). Upstream Kinase Analysis (UKA),^11^ a functional scoring method (PamGene) was used to rank kinases based on combined specificity scores (based on peptides linked to a kinase, derived from 6 databases) and sensitivity scores (based on treatment-control differences).

Statistical analysis

Data for healthy volunteers are shown as mean ± standard error of mean (SEM), whereas CLL patients are presented as median ± interquartile ranges. GraphPad Prism 8.3.0 software (La Jolla, CA, USA) was used for statistical analysis. For healthy donor and CLL patient samples a non-parametric and unpaired t-test (Mann-Whitney) was used to compare vehicle and inhibitor treatment in vitro, whereas for mouse samples statistics were calculated using a one-way non-parametric Anova (Kruskal-Wallis) to compare WT versus Btk-KO samples, as well as the comparison between vehicle and inhibitor treatment in both WT and Btk-KO mice. A p-value less than 0.05 was considered to be statistically significant in which * is p<0.05, ** is p<0.01 and *** is p<0.001.

References

1. Bergmeier, W, Bouvard, D, Eble, JA, Mokhtari-Nejad, R, Schulte, V, Zirngibl, H et al. Rhodocytin (aggretin) activates platelets lacking alpha(2)beta(1) integrin, glycoprotein VI, and the ligand-binding domain of glycoprotein Ibalpha. *J Biol Chem.* 2001; **276**, 25121-25126.

2. Rodeghiero, F, Tosetto, A, Abshire, T, Arnold, DM, Coller, B, James, P et al. ISTH/SSC bleeding assessment tool: a standardized questionnaire and a proposal for a new bleeding score for inherited bleeding disorders. *J Thromb Haemost.* 2010; **8**, 2063-2065.

3. van der Meijden, PE, Feijge, MA, Giesen, PL, Huijberts, M, van Raak, LP & Heemskerk, JW. Platelet P2Y12 receptors enhance signalling towards procoagulant activity and thrombin generation. A study with healthy subjects and patients at thrombotic risk. *Thromb Haemost.* 2005; **93**, 1128-1136.

4. Mattheij, NJ, Gilio, K, van Kruchten, R, Jobe, SM, Wieschhaus, AJ, Chishti, AH et al. Dual mechanism of integrin alphaIIbbeta3 closure in procoagulant platelets. *J Biol Chem.* 2013; **288**, 13325-13336.

5. Hendriks, RW, de Bruijn, MF, Maas, A, Dingjan, GM, Karis, A & Grosveld, F. Inactivation of Btk by insertion of lacZ reveals defects in B cell development only past the pre-B cell stage. *EMBO J.* 1996; **15**, 4862-4872.

6. de Witt, SM, Swieringa, F, Cavill, R, Lamers, MM, van Kruchten, R, Mastenbroek, T et al. Identification of platelet function defects by multi-parameter assessment of thrombus formation. *Nat Commun.* 2014; **5**, 4257.

7. van Geffen, JP, Swieringa, F, van Kuijk, K, Tullemans, BME, Solari, FA, Peng, B et al. Mild hyperlipidemia in mice aggravates platelet responsiveness in thrombus formation and exploration of platelet proteome and lipidome. *Sci Rep.* 2020; **10**, 21407.

8. van Geffen, JP, Brouns, SLN, Batista, J, McKinney, H, Kempster, C, Nagy, M et al. High-throughput elucidation of thrombus formation reveals sources of platelet function variability. *Haematologica.* 2019; **104**, 1256-1267.

9. Feijge, MA, van Pampus, EC, Lacabaratz-Porret, C, Hamulyàk, K, Levy-Toledano, S, Enouf, J et al. Inter-individual variability in Ca2+ signalling in platelets from healthy volunteers: effects of aspirin and relationship with expression of endomembrane Ca2+-ATPases. *Br J Haematol.* 1998; **102**, 850-859.

10. Jooss, NJ, De Simone, I, Provenzale, I, Fernandez, DI, Brouns, SLN, Farndale, RW et al. Role of Platelet Glycoprotein VI and Tyrosine Kinase Syk in Thrombus Formation on Collagen-Like Surfaces. *Int J Mol Sci.* 2019; **20**. 2788.

11. Chirumamilla, CS, Fazil, M, Perez-Novo, C, Rangarajan, S, de Wijn, R, Ramireddy, P et al. Profiling Activity of Cellular Kinases in Migrating T-Cells. *Methods Mol Biol.* 2019; **1930**, 99-113.
