# Supplementary material for: Comparison of inhibitory effects of irreversible and reversible Btk inhibitors on platelet function
Source: EJHaem. 2021 Aug 10;2(4):685–99. doi: 10.1002/jha2.269 (PMC9175945; doi:10.1002/jha2.269)
Supplement: Supplementary file 10 — Table S1 [file JHA2-2-685-s007.docx]

**Comparison of inhibitory effects of irreversible and reversible Btk inhibitors on platelet function**

Bibian M.E. Tullemans et al.

**Supplemental Table and Figures**

**Supplement Table 1:** IC_50_ values (μM) of Btk inhibitors for inhibition of GPVI- and CLEC-2 induced platelet aggregation and activation in presence or absence of plasma.

| **Btk inhibitor** | **Aggregation** | | | **Activation** | | |
| --- | --- | --- | --- | --- | --- | --- |
|  | **GPVI** | | **CLEC-2** | **GPVI** | | **GPVI + PAR** |
|  | PRP | WP | WP | Integrin activation | Secretion | PS  exposure |
| Ibrutinib | 3.31 | 0.425 | 0.170 | 0.196 | 0.176 | 0.332 |
| Acalabrutinib | 9.85 | 1.635 | 1.275 | 0.982 | 1.439 | 0.723 |
| MK-1026 | 8.84 | 0.820 | 0.487 | 0.388 | 0.807 | 0.410 |

PRP = Platelet-rich plasma, WP = Washed platelets, PS = phosphatidylserine

**Supplement figure 1: Platelet aggregation and activation of blood samples from healthy volunteers with ibrutinib and acalabrutinib, as well as dose-response of collagen-induced aggregation. (A)** Washed platelets (250x10^9^/L) from healthy volunteers were incubated with vehicle, ibrutinib (3.3 µM) or acalabrutinib (3.3 µM). Aggregation was induced by (i) 2MeS-ADP (1 µM), (ii) collagen type I (5 µg/mL), (iii) thrombin (1 nM) or (iv) U46619 (1 µM). Aggregation responses were measured during 8 minutes. **(B)** PRP (250x10^9^/L) was incubated with vehicle, ibrutinib (5 µM) or acalabrutinib (5 µM) for 10 minutes at 37°C and stimulated with ristocetin (2 mg/mL). Aggregation responses were measured during 8 minutes. **(C)** Washed platelets (100x10^9^/L) were stimulated with 2MeS-ADP (1 µM) or thrombin (1 nM) for 15 minutes and analysed by flow cytometry. Shown are percentages of platelets binding FITC-labelled PAC1 mAb against integrin α_IIb_β_3_ and AF647-labelled CD62P mAb for α-granule secretion, respectively. Data are shown as means ± SEM (n=5-6). * p<0.05, ** p<0.01, *** p<0.001 vs vehicle.

**Supplement figure 2:** **Thrombus formation under flow with blood from healthy donors in presence of acalabrutinib or ibrutinib over multiple microspots. (A)** Representative brightfield and fluorescence images and **(B)** quantification of control (vehicle, white bars), acalabrutinib (5 µM, grey bars) or ibrutinib (5 µM, black bars) over (i) collagen type I, (ii) collagen type III, (iii) vWF co-coated with rhodocytin, (iv) vWF co-coated with laminin, (v) vWF co-coated with ristocetin and (vi) vWF co-coated with fibrinogen. P1 = morphological score of platelet adhesion and thrombus formation, P2 = surface area coverage of adhered platelets, P3 = platelet aggregate contraction score, P4 = platelet aggregate multilayer score, P5 = coverage of multi-layered platelet aggregation, P6 = integrin α_IIb_β_3_ activation, P7 = P-selectin expression and P8 = PS exposure. Data are shown as means ± SEM (n=6-8). * p<0.05, ** p<0.01, *** p<0.001.

**Supplement figure 3: Thrombus formation and platelet activation with blood of WT or Btk-KO mice.** Citrated whole blood from WT or Btk-KO mice pre-incubated with control (vehicle, white bars), acalabrutinib (5 µM, grey bars) or ibrutinib (5 µM, black bars) was perfused for 3.5 minutes at a wall shear rate of 1,000 s^-1^ over a collagen type I surface. **(A)** Quantification of brightfield and fluorescence images. **(B-C)** Platelet activation in whole blood induced by **(B)** 2MeS-ADP (1 µM) or **(C)** PAR4AP (100 µg/mL). Histograms show the percentages of platelets binding PE-labelled JON/A monoclonal antibody or PerCP-labelled CD62P mAb in WT and Btk-KO mice. Data are presented as means ± SEM (n=5-6), * p<0.05, ** p<0.01 vs vehicle or indicated otherwise.

**Supplement figure 4: Platelet aggregation and activation of blood samples from healthy volunteers in presence of MK-1026. (A)** Washed platelets (250x10^9^/L) from healthy volunteers were incubated with vehicle or MK-1026 (3.3 µM). Aggregation was induced by (i) 2MeS-ADP (1 µM), (ii) collagen type I (5 µg/mL), (iii) thrombin (1 nM) or (iv) U46619 (1 µM). Aggregation responses were measured during 8 minutes. **(B)** PRP (250x10^9^/L) from healthy volunteers were incubated with vehicle or MK-1026 (5 µM) for 10 minutes at 37°C and stimulated with ristocetin (2 mg/mL). Aggregation responses were measured during 8 minutes. **(C)** Washed platelets (100x10^9^/L) were stimulated with 2MeS-ADP (1 µM) or thrombin (1 nM) for 15 minutes and analysed by flow cytometry. Shown are percentages of platelets binding FITC-labelled PAC1 mAb against integrin α_IIb_β_3_ and AF647-labelled CD62P mAb for α-granule secretion, respectively. Data are represented as means ± SEM (n=5-6), * p<0.05.

**Supplement figure 5: Thrombus formation under flow with blood from healthy volunteers in presence of MK-1026 over multiple microspots.** **(A)** Representative brightfield and fluorescence images and **(B)** quantification of control (white bars), MK-1026 (sand-coloured bars) over (i) collagen type I, (ii) collagen type III, (iii) vWF co-coated with laminin, (iv) vWF co-coated with rhodocytin, (v) vWF co-coated with ristocetin and (vi) vWF co-coated with fibrinogen. Data are shown as means ± SEM (n=6).

**Supplement figure 6: Thrombus formation under flow with blood of WT or Btk-KO mice over collagen type I surface. (A)** Quantification of brightfield and fluorescence images of control (white bars) or MK-1026 (sand-coloured bars) over collagen type I. Parameter 1-8 are as described for Supplement Figure 2. **(B-C)** Platelet activation in whole blood induced by **(B)** 2MeS-ADP (1 µM) or **(C)** PAR4AP (100 µg/mL). Histograms show the percentages of platelets binding PE-labelled JON/A monoclonal antibody or PerCP-labelled CD62P mAb in WT and Btk-KO mice. Data are presented as means ± SEM (n=5-6), * p<0.05, ** p<0.01 vs vehicle or indicated otherwise.

**Supplement figure 7: Thrombus formation under flow with blood samples of patients with or without ibrutinib treatment.** Citrated whole blood from healthy controls and patients (with or without ibrutinib treatment) was perfused for 3.5 minutes at a wall shear rate of 1,000 s^-1^ over coated microspots. Quantification of brightfield and fluorescence images of healthy donors (white bars), patients (light grey bars) or patients receiving ibrutinib (black bars) over **(A)** collagen type I, **(B)** vWF co-coated with rhodocytin and **(C)** vWF co-coated with laminin. Parameter 1-8 are as described for Supplement Figure 2. Data are presented as medians ± interquartile ranges (n=6-9), * p<0.05, ** p<0.01, *** p<0.001 vs patient without treatment.

**Supplement figure 8: Thrombus formation and aggregation with blood samples of ibrutinib-treated patients with or without bleeding.** Citrated whole blood from patients receiving ibrutinib treatment was perfused for 3.5 minutes at a wall shear rate of 1,000 s^-1^ over coated microspots. Quantification of brightfield and fluorescence images of ibrutinib-treated patients with a ISTH BAT score of 0-1 (black dots) or a ISTH BAT score over a value of 2 (red dots) over **(A)** collagen type I, **(B)** vWF co-coated with rhodocytin and **(C)** vWF co-coated with laminin. Parameters 1, 6-8 are as described for Supplement Figure 2. **(D)** Light transmission aggregometry was induced in isolated platelets (250x10^9^/L) by collagen (1 µg/mL), rhodocytin (1 µg/mL), 2MeS-ADP (1 µM), U46619 (1 µM) or thrombin (1 nM). Scatter plots show the percentage of aggregation of patients receiving ibrutinib with a low bleeding score (0-1, black dots) or a bleeding score of 2 or higher (red dots). Each dot represents one individual patient. Data are presented as medians ± interquartile ranges (n=3-6).
